# Acknowledgement to Reviewers of *International Journal of Molecular Sciences* in 2016

**DOI:** 10.3390/ijms18010136

**Published:** 2017-01-11

**Authors:** International Journal of Molecular Sciences Editorial Office

**Affiliations:** MDPI AG, St. Alban-Anlage 66, 4052 Basel, Switzerland; ijms@mdpi.com

The editors of *International Journal of Molecular Sciences* would like to express their sincere gratitude to the following reviewers for assessing manuscripts in 2016.

We greatly appreciate the contribution of expert reviewers, which is crucial to the journal’s editorial process. We aim to recognize reviewer contributions through several mechanisms, of which the annual publication of reviewer names is one. Reviewers receive a voucher entitling them to a discount on their next MDPI publication and can download a certificate of recognition directly from our submission system. Additionally, reviewers can sign up to the service Publons (https://publons.com) to receive recognition. Of course, in these initiatives we are careful not to compromise reviewer confidentiality. Many reviewers see their work as a voluntary and often unseen part of their role as researchers. We are grateful to the time reviewers donate to our journals and the contribution they make.

If you are interested in becoming a reviewer for *International Journal of Molecular Sciences*, see the link at the bottom of the webpage http://www.mdpi.com/reviewers.

The following reviewed for *International Journal of Molecular Sciences* in 2016:
Abbott, Karen L.Hervás-Pérez, J.P.Peng, Sheng-BinAbdelmohsen, KotbHess, AngelaPeng, Wen-HuangAbdel-Wahab, OmarHetman, MichalPennypacker, KeithAbdulah, RizkyHettiarachchi, D.S.Pentzold, StefanAbe, SumikoHettinga, KasperPeppelenbosch, MaikelAbel, PeterHewett, James A.Perán, MacarenaAbenavoli, LudovicoHeyn, PatriciaPerazzolli, MicheleAbizaid, AlfonsoHiblot, JulienPercival, SusanAblonczy, ZsoltHicks, AlexanderPereira, C.Abraham, Wolf-RainerHicks, Glenn R.Pereira, DavidAbranches, RitaHidalgo-Cantrabana, ClaudioPereira, Olívia R.Abriata, Luciano A.Hider, RobertPereira, SusanaAburima, AhmedHigashi, TomomiPerepelyuk, MarynaAchinger-Kawecka, JoannaHiggins, ClairePerera, Omaththage P.Achsel, TilmannHiguchi, AkonPerez-Cano, FranciscoAdachi, HiroakiHildebrand, MarkPérez-Castrillón, José L.Adamakis, I.-D.S.Hildt, EberhardPérez-Correa, José RicardoAdamcakova-Dodd, AndreaHill, William G.Pérez-Jiménez, A.J.Adamo, VincenzoHiller, MonikaPerez-Jimenez, JaraAdams, JamesHiltermann, T. Jeroen N.Pérez-Montaño, FranciscoAdegoke, O.A.J.Hiltner, Jana KatharinaPerez-Stable, CarlosAdoro, StanleyHilz, MaxPéros, Jean-PierreAdunyah, Samuel E.Hingorani, DinaPerrais, MichaëlAfantitis, AntreasHiraga, ToruPerreault, JonathanAfrin, SadiaHirakawa, HidehikoPerricone, CarloAgarwal, NeerajHirano, KatsuyaPerrier, JosetteAgger, Jane W.Hirano, ShigeruPerry, Mark M.Agodi, AntonellaHirasawa, NoriyasuPersson, RasmusAgon, VanessaHirasawa, TakashiPesis, EdnaAgrawal, AlokHiroaki, FujiiPetersen, BjörnAgrawal, Sandeep K.Hirohashi, YoshihikoPeterson, DanielAgrimson, KellieHirotsune, ShinjiPeterson, Eliza J.R.Agulló-Ortuño, M. TeresaHirsch, PierrePeterson, SteveAgut, MontserratHleap, Jose SergioPetitot, Anne-SophieAhi, YadvinderHo, Roger Chun-ManPetropoulos, IsabelleAhmad, ZeeshanHoeijmakers, Janneke G.J.Petrosyan, ArmenAhmed, Ammar M.Hoemann, Caroline D.Pettersen, CarolineAhmed, NisarHoene, MiriamPettit, AllisonAhrens, ChristianHoffmann, GeorgPeulen, OlivierAikawa, TakuyaHöfinger, SiegfriedPezzella, AlessandroAires, AlfredoHofmann, JohannPfeffer, LarryAiroldi, CristinaHofmann, Thomas G.Phanstiel, OttoAkahane, ManabuHojjat-Farsangi, MohammadPhatarpekar, PrasadAkamatsu, MikiHolder, AlvinPhelps, Patricia E.AKASHI, MakotoHolland, GregoryPhilips, NeenaAkdemir, DenizHolliday, L. ShannonPhillips, Jacqueline K.Akitsu, TakashiroHöllig, AnkePhillips, James B.Akopian, Armen N.Hollman, Peter C.H.Phillips, Robert S.Alam, AntoineHollomon, Mario G.Phillips-Jones, Mary K.Alander, JarmoHolmes, Dawn E.Phipps, Richard P.Alary, FabienneHolmes, GregPi, LiyaAlavi, SamanHolt, Scott M.Piazza, FabrizioAlbahrani, Ali A.Höltke, CarstenPicariello, GianlucaAlbano, EmanueleHolton, TheresePiccialli, GennaroAlbericio, FernandoHolzinger, MichaelPichler, MartinAlberti, SaverioHolzmann, NicolePickett, Stephen D.Albinsson, SebastianHomem, RafaelPickova, JanaAl-Dujaili, EmadHondan, TomoyukiPicorel Castaño, RafaelAlenghat, Francis J.Hong, Kyeong-ManPidko, Evgeny A.Alessandro, RiccardoHong, Yeong HoPierini, AntonioAlexandre-Gouabau, M.-C.Hong, YonggeunPiggins, Hugh D.Alexiou, ChristophHong, YunhanPignata, ClaudioAlfaidy, N.Hönicka, MarkusPignataro, GiuseppeAlfano, DanielaHonoré, Patrick M.Pignolo, Robert J.Alfano, MassimoHooper, Antony MarkPiiper, AlbrechtAlfonzo, AntonioHordvik, IvarPikart, Tiago GeorgAlfredsson, JennyHorsefield, SusannaPilati, StefaniaAl-Horani, RamiHorvath, David P.Pilishvili, TamaraAliev, FazilHoshi, AkihikoPilkington, Suzanne M.Alkilani, Ahlam ZaidHotta, AkitsuPillay, VinessAllegaert, KarelHottiger, MichaelPilon-Thomas, ShariAlmajano, María PilarHou, Chien-WeiPineault, NicolasAlmasio, Piero LuigiHou, HarveyPineda, MiguelAlmstrup, KristianHou, Yueh-JuPinto, AntonioAloisi, Anna MariaHouen, GunnarPinzani, PamelaAlonso, ConchitaHoughton, DavidPiomboni, PaolaAlonso-Alconada, DanielHouston, Kevin D.Pioszak, AugenAlpini, GianfrancoHouston, Mark C.Piróg, Katarzyna A.Alsaweed, MohammedHouston, RossPirola, LucianoAlsteens, DavidHribal, MartaPisabarro, Antonio G.Alt, David P.Hrobárik, PeterPisani, AntonioAlteri, Christopher J.Hsia, Shih-MinPisitkun, TrairakAltintas, ZeynepHsiao, GeorgePislariu, CatalinaAluwini, ShafakHsieh, Nan-HungPisoni, Giorgia BrambillaAlvarez, CarlosHsieh, Pei-WenPitto, LetiziaAlvarez-Suarez, José MiguelHsieh, Yi-HsienPitto-Barry, AnaïsÁlvaro, TomásHsin, Kun-YiPiva, TerrenceAlves, CelsoHsu, DanielPivetal, JérémyAlves, SandraHsu, Li-SungPixley, SarahAlwahsh, Salamah M.Hsu, ToddPizzo, ElioAlyokhin, AndreiHu, Michael Sung-MinPlantinga, TheoAlzaid, F.Hu, NingPlatt, Jeffrey L.Amant, CaroleHu, Shao-YangPlatts, JamesAmar, LaurenceHu, YinlingPlaza, Gustavo R.Amaral, AlexandraHu, YuPlazzi, FedericoAmasheh, SalahHuang, A-MeiPliego-Alfaro, FernandoAmbe, KimiharuHuang, Chi-ChangPlock, Jan A.Ambe, Peter C.Huang, Chih-LingPloughman, MichelleAmbigapathy, GaneshHuang, Chiu-JungPochynyuk, OlehAmicarelli, FernandaHuang, Genin GaryPoddar, RanjanaAmital, HowardHuang, HaoPodszun, MarenAmmendola, RosarioHuang, Hsuang-TingPoelstra, JelmerAmorati, RiccardoHuang, JianPoinsot, VerenaAmorós, AsunciónHuang, JianguoPoirier, Guy G.Anczukow-Camarda, OlgaHuang, RenhuaPoirier, Raymond A.Andersen, BogiHuang, Shih-YiPoirot, MarcAndersen, MortenHuang, Tzou-ChiPoldermans, DonAndersen, Thomas LevinHuang, Wen-ChinPoleszak, EwaAnderson, Anne J.Huang, Wen-LiiPolette, MyriamAnderson, Travis M.Huang, WenlinPoli, AlessandroAndersson, Håkan SHuang, YutingPoli, GuidoAndersson, Leif C.Huang, Zhan-PengPollitt, KrystalAndersson, Mats X.Huang, ZhiPolticelli, FabioAndrade, FernandoHuangfu, DanweiPolyak, Stephen J.Andrade, PaulaHuchzermeyer, BernhardPommer, BernhardAndré, VâniaHuelsen, TimPonnambalam, SreenivasanAndrea, OnofriHuen, Michael Shing YanPons, AntoniAndreadou, IoannaHuertas-Pérez, José FernandoPons, TirsoAndré-Fontaine, GenevieveHughes, Timothy C.Popa-Wagner, AurelAndreoni, FrancescaHuh, Jin HoePopescu, NirvanaAndreozzi, Giuseppe MariaHuhn, RagnarPorpora, Maria GraziaAndres, Allen M.Hui, YePorteu, FrançoiseAndrukhov, OlehHuleihel, MahmoudPosé, DavidAnes, ElsaHulten, Kristina G.Post, JanineAngeletti, MauroHung, Jan-JongPotterat, OlivierAngelico, FrancescoHunsicker-Wang, Laura M.Pouliot, NormandAngelini, ClaudiaHunt, Piper ReidPoulsen, AndersAngelone, TommasoHunter, Wayne B.Pountney, DeanAngelucci, AdrianoHuppi, KonradPourahmad, JalalAngulo, JavierHuq, LailaPourquier, PhilippeAniello, FrancescoHur, WonheePowell, DanielAnifandis, GeorgeHura, TomaszPowers, Evan T.Annunziata, PasqualeHurst, Douglas R.Pozo, Óscar J.Anraku, MakotoHursthouse, AndrewPozzobon, MichelaAnsorena, DianaHusnjak, KoraljkaPrante, OlafAnsquer, Jean ClaudeHüttemann, MaikPrats, Anne-CatherineAntaris, Alexander LeeHwang, Bang YeonPratsinis, HarrisAnthony, KarenHwang, Dae YounPrchal, JosefAntognelli, CinziaHwang, Eun SookPreckel, BenediktAntoni, DelphineHwang, Ming-JingPreissner, Klaus T.Antoniadi, IoannaHwang, William Ying KheePrentice, HowardAntoniak, SilvioHwang, Woo SukProctor, Richard A.Aon, Miguel A.Hyun, Jin WonProestos, CharalamposAparicio, FredericHyun, Sang-HwanProudfoot, Amanda E.I.Aperia, AnitaIacobellis, GianlucaProvost, PatrickAppetecchi, G.B.Iadarola, PaoloPruszyński, MarekAprea, EugenioIadevaia, ValentinaPrykhodko, OlenaApte, SwapnaIannuccelli, ValentinaPu, ChristyApte, UdayanIasevoli, FelicePu, JingArai, RyoichiIbarra Molero, BeatrizPucci, IdaAraniti, FabrizioIbrahim, HussameldinPuddu, Paolo EmilioArasaradnam, Ramesh P.Ibrahim, ToniPuglia, DeboraArbefeville, SophieIchikawa, HiroshiPuglisi, FabioArbona, VicentIchimura, HiroshiPühler, AlfredArcher, TrevorIebba, ValerioPuiggalí, J.Archibald, SteveIentile, RiccardoPuiggalí, JordiArcidiacono, BiagioIglesias, EmiliaPulati, NuerxidaArdley, JulieIguchi, A.Pulido, DavidArduini, AlessandroIhalainen, Teemu O.Pulignani, SilviaArens, PaulIhle, AndreasPulinilkunnil, ThomasArese, MarziaIida, HiroshiPulliainen, ArtoAriani, AndreaIinuma, HisaePumera, MartinAriga, KatsuhikoIkai, TomoyukiPuri, AkshitArnhold, JuergenIkeda, HajimePutz, Mihai V.Aroca, RicardoIkeda, MasahiroPuvvula, Pavan KumarArpawong, Thalida E.Ikeshima-Kataoka, HirokoQaderi, Mirwais M.Arrick, DeniseIlardi, VincenzaQi, Robert Z.Arrighi, SilvanaIlla, MiriamQi, XinArroyo, CristinaImai, KazushiQian, LiArthur, MichelImajoh, MasayukiQian, PengxuArtlip, TimothyImig, JochenQian, WeiArul, JosephImmel, FrançoiseQin, Xue-MeiAsa, Sylvia L.Inaba, HiroshiQiu, HuanAsahina, KinjiInagaki, NaokiQiu, JianwenAsakura, TetsuoIndo, Hiroko P.Qiu, WeiAsano, RyutaroIndriolo, EmilyQuail, Daniela F.Asard, HanInman, SharonQuan, GuoxingAscher, David B.Inokuchi, TsutomuQuan, TaihaoAsensi, V.Inoue, AtsukoQuesada, VíctorAshiuchi, MakotoInoue, G.Quigley, EamonAsimakopoulos, A.G.Inoue, YasumichiQuigley, EamonnAssfeld, XavierInsel, Kathleen C.Quillard, ThibautAstolfi, StefaniaInselman, AmyQuinn, JulianAtanasov, Atanas G.Inta, DragosQuintana, JoséAtanassova, RossitzaInui, HideyukiQuintana, M.C.Atangana, Alain R.Inui, MasafumiRachek, LyudmilaAtes-Alagoz, ZeynepInuzuka, HiroyukiRachmawati, DessyAthyros, VasiliosIolanda, FrancoliniRacioppi, LuigiAtkinson, SarahIorio, EgidioRad, BehzadAtluri, Venkata S.R.Iorio, RaffaeleRadhakrishnan, RaviAtrian, SilviaIorizzo, MassimoRådinger, MadeleineAtsawasuwan, PhimonIraci, NunzioRadner, FranzAttanasio, ChiaraIrakli, MariaRadons, JurgenAttardo, Geoffrey M.Irato, PaolaRadwan, Osman E.Audet, CélineIrina, MoreiraRaff, JohannesAuguet, TeresaIrjala, HeikkiRaffaelli, MarcoAury-Landas, JulietteIsaji, ShujiRaghupathi, WullianallurAustin, BrianIsemura, MamoruRagsdale, DavidAustin, CarolineIshibashi, KenichiRagusa, Maria AntoniettaAutelli, RiccardoIshii, IsaoRahimi, FaridAvdic, SelmirIshikawa, YasukoRahman, ZiaurAverbeck, DietrichIshitsuka, YoichiRai, Padmalatha S.Avola, R.Ishizaki, TakumaRaimondi, LaviniaAvrova, Natalia F.Islam, M. NurulRajaputra, PallaviAylon, YaelIslam, Md SorifulRajarathnam, KrishnaAyoub, Nadia A.Isman, MurrayRajasekaran, ParthibanAyyavoo, VelpandiIsokpehi, RaphaelRajkkumar, PremrajAzarbal, Amir F.Ito, ShigekiRajput, AshwaniAzarnia Tehran, DomenicoIto, ShosukeRakofsky, Jeffrey J.Azevedo, HelenaIto, YasuhiroRakotondrafara, AurelieAzmi, Asfar SohailItoh, KazuyukiRakus, DariuszAzoitei, NinelItoh, MasayukiRama, PaoloAzuma, MasaakiItoh, ToshiyukiRamachandran, SrinivasanBaba, HideoItoh, YoshifumiRamadan, QasemBabajko, SylvieItou, JunjiRamakrishna, SureshBabu, AnishIvankin, AndreyRamalingam, LathaBabu, DineshIvanov, AlexanderRaman, SripriyaBaburamani, AnaIwaizumi, Masakazu GRamani, KomalBache, SørenIwamori, MasaoRamani, N.V.Backus, DeborahIwamoto, SadahikoRamaraj, PanduranganBae, Yoe-SikIwashima, YoshioRamidi, PunnamchandarBaechlein, ChrsitineIzquierdo-Useros, NuriaRamírez, José M.Baeg, Gyeong HunIzumi, KoujiRamis, RebecaBaer, Patrick C.Izumi, NamikiRamón, DanielBagby, MichaelIzumiya, YoshihiroRamonda, RobertaBagni, ClaudiaJack, ThomasRamos, SoniaBai, Li-YuanJackson, ChristopherRamos-Morales, FranciscoBai, WeiJackson, Miriam T.Ramos-Vivas, JoséBailard, Neil S.Jackson, StephenRamzi, MohammedBairaktari, Eleni T.Jacob, Christoph R.Ran, ChongzhaoBaisantry, ArpitaJacob, FrancisRana, DipakBajpai, RichaJacobsen, Elisabeth EgholmRand, MatthewBaker, Simon C.Jacot, WilliamRandall, Stephen K.Baksh, ShairazJacquin, LisaRanganathan, NatarajanBalandraud, NathalieJaeschke, HartmutRanieri, ElenaBalatti, PedroJagerovic, NadineRanzato, EliaBalcells, MercedesJagiello, KarolinaRao, C.V.Baldacci-Cresp, FabienJaimovich, EnriqueRao, RosaBaldermann, SusanneJaiswal, Ashvin RRaposo, Maria Filomena JesusBaldoni, ElenaJaiswal, J.K.Rappaport, JayBalduini, WalterJakob, Stephan M.Rasche, LeoBaldwin, ScottJakočiūnas, TadasRaspanti,, MarioBalestrini, Raffaella MariaJakowski, Joseph D.Rassoulzadegan, MinooBalietti, MartaJalkanen, SirpaRastegar, SepandBall, JonathanJames, EuanRatcliffe, R. GeorgeBallerini, ClaraJames, Nicholas G.Raterman, H G RatermanBallestri, StefanoJan, Fuh-JyhRaucher, DrazenBallottari, MatteoJang, Ik-SoonRavegnini, GloriaBaltazar, FátimaJang, Jer-HuanRavid, KatyaBaluska, FrantisekJang, KiseokRawashdeh, OliverBamford, ConnorJang, SeonghoeRawel, HarshadraiBanack, SandraJang, WooyoungRawls, Henry R.Banchero, MauroJann, Michael W.Ray, ParthaBanerjee, AreenJansen, EugenRay, RanjitBanerjee, Hirendra NathJanssen, ArneRaynes, J.K.Banerjee, JheelamJarausch, WolfgangRazumilava, NataliyaBanerjee, KalpitaJarret, Robert L.Rea, KatiaBanerjee, PratikJärvenpää, Eila P.Reading, Benjamin J.Baniahmad, AriaJarvis, Gary A.Reale, MarcellaBanilas, GeorgiosJat, Parmjit S.Rebelo, IreneBanskota, Arjun H.Jauch, RalfRebl, AlexanderBanti, Christina N.Jaumot, JoaquimRebollar, EstherBao, GuanhuiJawien, JacekRedden, RobertBapputty, ReenaJayaraman, DhileepkumarReddy, Narsa M.Baptiste-Roberts, KeshaJayasinghe, SuwanReddy, SakamuriBarabutis, NektariosJay-Gerin, Jean-PaulReddy, VenkateshwarBaratta, FrancescoJay-Russell, MicheleRedell, MicheleBarba, FranciscoJazirehi, AliRedon, Christophe E.Barba, MaddalenaJendelova, PavlaReeve, AmyBarbagallo, DavideJennings, Lance C.Reeve, VivienneBarbas, CoralJensen, EricRegenbrecht, Christian R.A.Barbayianni, EfrosiniJensen, L.H.Rehrig, Erin M.Barberi, TizianoJensen, Randy L.Reid, DugaldBarbier, OlivierJensen, ThomasReijns, MartinBarbieri, FedericaJeong, Byoung RyongReinach, Peter S.Barbosa-Leiker, CelestinaJeong, DaewonReinders, JoergBariana, HarbansJeong, JeeyonReindl, MarkusBarnea, Eytan R.Jeong, Keun-YeongReineke, AnnetteBarone, FlaviaJeong, Seon-YongReinhardt, DirkBarradas, DidierJeong, Won-JoongReisman, Scott A.Barreira, LuísaJeong, Woo-SikReissmann, SiegmundBarros, PedroJeschke, UdoRemuzzi, GiuseppeBar-Shavit, RachelJesionowski, TeofilRen, JianguoBarta, CsengeleJetten, AntonRen, NatalieBartfeld, SinaJeyaseelan, KandiahRen, ShuxinBartha, IstvánJeyasekharan, AnandRenaudineau, YvesBartoli, FrancescoJha, AmitRendeiro, CatarinaBarzilay, Joshua I.Jheon, SanghoonRenner, UlrichBasak, DebasishJi, DebinRennert, C.Basaraba, Randall J.Jia, BaoleiRenshaw, Stephen A.Basavarajappa, BalapalJia, LeiRenzoni, ElizabethBashan, YoavJiang, MeiReshkin, Stephan JoelBasini, GiuseppinaJiang, QingRevelli, LucaBassil, Nahla V.Jiang, Shu-YeRevilla, EugenioBasso, KariJiang, WeiRex, ToniaBastola, Dhundy R.Jiang, XiaohuaRey, A.I.Basu, AbhijitJickling, GlenRey, PatriceBasu, SutapaJiménez-Araujo, AnaRezzani, RitaBatail, PatrickJiménez-Cervantes, CeliaRhazi, LarbiBates, DarcyJin, Dong-HoonRhee, Ki-JongBattaglia, AgatinoJin, GeRialland, MickaëlBattistelli, MichelaJin, HuajieRiazuddin, S.Bauer, MartinJin, LiRibeiro, IsabelBaum, ChristelJin, XinRibeiro, RogerioBaum, JamieJing, FuyuanRibeiro, SusanaBaum, MichelJob, DominiqueRibeiro-Barros, Ana I.Baumann, ArndJoensuu, Jussi J.Ricciardelli, CarmelaBaumann, HeinzJoglekar, MadhuraRich, AlisonBaumgärtner, WolfgangJohn, RohanRichards, Renée StirlingBavilacqua, AntonioJohnson, Arlen W.Richardson, AlanBayliss, SionJohnson, Gregory A.Richardson, Mark F.Bazhin, AlexandrJohnson, Jason L.Rider, CynthiaBazylińska, UrszulaJohnson, Paul D.Rieder, MichaelBecana, ManuelJohnson, Thomas V.Riedl, RainerBecerra, S. PatriciaJohnston, G. PatriciaRief, HaraldBechmann, IngoJohnston, MichaelRieg, Timo M.Beck, DominikJoles, JaapRieger-Christ, Kimberly M.Beck, WilliamJolkkonen, JukkaRiess, OlafBecker, ChristophJones, AngusRigas, StamatisBeckmann, ManfredJones, BrianRimondini, LiaBecuwe, PhilippeJones, DavyRimstad, EspenBede, Jacqueline C.Jones, HuwRinaldo, Charles R.Bedia, CarmenJones, JohnRink, CameronBedini, EmilianoJones, Rachel A.Rinnerthaler, GabrielBednarski, PatrickJones, T. BuckyRiou, Laurent M.Beggs, SimonJonnalagadda, Subash C.Risk, Janet M.Behera, SmrutisanjitaJoo, Choun-KiRittner, HeikeBeilby, MaryJordao, Antonio M.Ritze, YvonneBeitz, EricJörg, FrederikeRo, Simon WeonsangBela-Ong, DennisJørgensen, Anders PalmstrømRoan, Nadia R.Beldi, GuidoJørgensen, MaleneRoato, IlariaBelkacemi, LouizaJose, Pedro A.Roberto, VâniaBell, StephenJoseph, ShannonRoberts, AshleyBella, FedericoJoshi, BharatRobey, PamelaBellés, José MaríaJouanneau, SulivanRobinson, BrooksBellezza, IlariaJoubert, MichaelRobledo-Sierra, JairoBello, SalvadorJouneau, AliceRobson, KathrynBello, Xanel VecinoJoußen, NicoleRoccheri, Maria CarmelaBellomo, Elisa A.Jovanović, AleksandarRocha, SoniaBelmadani, SouadJoven, JorgeRocha-Ferreira, E.Beloqui, AnaJover, RamiroRoche, DanielBelstrøm, DanielJoyce, Susan A.Rödel, FranzBelyaeva, NataliaJuang, Horng-HengRodicio, María CelinaBendahhou, SaïdJuarranz, ÁngelesRodino-Klapac, Louise R.Benedetti, MicheleJujić, AmraRodnina, MarinaBenediktsdóttir, Berglind EvaJuliano, ClaudiaRodrigo, IsmaelBeney, LaurentJulier, BernadetteRodrigues, Thaís BarrosBénichou, SergeJun, Ho-WookRodríguez, AmaiaBenito, Juan M.Jung, EunsunRodriguez, Cristina P.Benjak, AndrejJung, HyungtaekRodríguez, Manuel JoséBenoist, HervéJung, KlausRodríguez-Couto, SusanaBenoit, Danielle S.W.Jung, SeunhoRodríguez-Pascual, FernandoBen-Shimol, ShalomJung, SophieRodriguez-Revenga, LaiaBenson, Al B.Jung, Yong WooRoehe, RainerBenzeroual, Kenza E.Jung, Young MeeRoelen, BernardBerchtold, MartinJunker, KerstinRoesch, FrankBercion, SylvieJurjus, Rosalyn A.Roessner, UteBeretta, GiovanniJuszczak, LesławRoffler, Steve R.Bergen, WernerJyh-Fei, LiaoRogers, HilaryBerger, Kenneth I.Kabbani, Toufik A.Rogister, BernardBerkowitz, Bruce A.Kaczor, AgnieszkaRoh, ChanghyunBerland, SophieKadib, Abdelkrim ElRoh, SanghoBerlanga, Ángel MéridaKadokawa, Jun-ichiRohira, AartiBerlin, K. DarrellKaeffer, BertrandRomalde, JesusBermudez, MarcelKageyama, YukioRomano, FedericaBernardi, Rafael C.Kahn, MichaelRomanov, VictorBernards, MattKai, MihokoRomero Martínez, ÁngelBernasconi, PiaKaira, KyoichiRomero, Francisco JavierBernt, KathrinKaji, HiroshiRomero, Maria-PazBernt, MatthiasKaji, ToshiyukiRommer, PaulusBerron, BradKajiya, HiroshiRompel, AnnetteBertino, Massimo F.Kakinuma, SeiRoncada, PaolaBertolini, MartaKakinuma, YoshihikoRondanino, ChristineBertoni, GiuseppeKakisis, John D.Rong, YueguangBertram, RalphKako, TetsuyaRoos, JessicaBesada, CristinaKakulas, FoteiniRoque, CláudioBesalú, EmiliKalendar, RuslanRos Lis, Jose VicenteBesson, ThierryKalfakakou, VasilikiRosado, JuanBethea, Cynthia L.Kalinin, StanislavRosario, KarynaBetoret, EsterKaller, MarkusRose, Aaron H.Bettaieb, AliKallunki, TuulaRose, Alan B.Bettington, MarkKalogiannis, Konstantinos G.Rose, Christopher F.Bevilacqua, AntonioKalogirou, CharisRoseiro, Luísa B.Bhagwat, NikhilKamagata, KiyotoRosell, RafaelBhat, ShridharKamal, Abu Hena MostafaRosellini, DanieleBhattacharyya, GargiKambe, TaihoRoselló, SalvadorBhatwadekar, Ashay D.Kamihira, MasamichiRosenberg, HeleneBhonde, Ramesh RamchandraKamiloglu, SenemRosendahl, JonasBhutoria, SavitaKaminskyj, SusanRosenmann, HannaBi, EnguangKamioka, HiroshiRosenthal, PhilipBiasini, EmilianoKamitani, ShigekiRosenwasser, Alan M.Bickle, MarcKamiya, HidekiRosenzweig, DerekBid, Hemant K.Kamiya, KatsumasaRosi, AntonellaBiddle, AdrianKamp, ChristelRoss, SharonBiegstraaten, MariekeKamp, David W.Rossello, ArmandoBienert, Gerd P.Kamphuis, Lars G.Rossi, SimonaBiernasiuk, AnnaKamunde, CollinsRossignol, JulienBiersack, BernhardKan, CasinaRostaing, LionelBilbao, Jose RamonKanaoka, MasahiroRouhana, LabibBill, Roslyn M.Kane, Maureen A.Routier, Françoise H.Birch, JohnKang, Bum-YongRovaris, MarcoBirrell, MarkKang, Byoung-CheorlRovida, ElisabettaBishop, KarenKang, ChenRowland, IanBisht, VikramKang, ChulhunRowland, LisaBita, Craita EKang, HunseungRoy, JérômeBitter, IstvanKang, Jae SeungRoy, NilotpalBix, GregoryKang, JaekuRoy, Peter J.Bizzarri, MarianoKang, JeehoonRoy, SantanuBjörkbacka, HarryKang, JonghoonRoy, SomakBjörnsson, EinarKang, LeiRoy, SouravBlackburn, Daniel G.Kang, Min-JungRoychowdhury, SanjoyBlagden, SarahKang, Sang SunRozhkova, ElenaBlair, PaulKang, SungminRozhon, WilfriedBlais, AnneKang, Taek WonRuan, XiangboBlaker, JonnyKang, WeiRubakhin, StanislavBlamires, Sean J.Kang, WonkuRubert, Josep RubertBlanco, José Manuel LopezKango-Singh, MadhuriRudkin, BrianBlandino, GiovanniKanvil, SadiaRudyk, OlenaBlando, FedericaKanzaki, HiroyukiRuff, AdrianBlank, MichaelKao, Erl-ShyhRuggeri, PaoloBłaszczyk, LidiaKapfhammer, JosefRuggerone, PaoloBlattner, C.Kaplan, Barry B.Ruiu, LucaBleackley, Mark R.Kappen, Jasper H.Ruiz, JoséBlesso, ChristopherKarakasidis, T.E.Ruiz, Oscar A.Bloemers, JosKararigas, GeorgiosRuiz, T.Board, MaryKärenlampi, SirpaRuiz-Echevarria, Maria J.Bobe, RégisKarginov, FedorRund, DeborahBoccuto, LuigiKarkhanis, VrajeshRunte, ChristophBock, C.-ThomasKarki, RajendraRuppert, J. MichaelBode, Robert F.Karlic, HeidrunRupprecht, RainerBodenstine, ThomasKarlsson, OskarRuseva, MarietaBodnar, RichardKårlund, AnnaRush, Demaretta S.Boeckh-Behrens, T.Karmakar, ParthaRusnati, MarcoBoelsterli, Urs A.Karras, S.N.Ruso, JuanBoer, Judith M.Kasahara, HidekoRuss, BrendanBogan, Jonathan S.Kaschina, ElenaRussell, Fraser D.Bögemann, MartinKashman, YoelRussell, JoanneBogni, AlessiaKashuba, ElenaRusso, AntonioBogomolnaya, Lydia M.Kasoju, NareshRusso, MariaBoguszewicz, Ł.Kassiri, ZamanehRusso, Matteo A.Bøgwald, JarlKasten-Jolly, JaneRussu, Wade A.Böhm, VolkerKatam, RameshRutgers, MichielBohutskyi, PavloKataoka, NaoyukiRüther, ThomasBohutskyi, PavloKatapodis, PetrosRuthstein, SharonBoisvert, François-MichelKatayama, ShigeruRutkowski, JosephBoka, Vasiliki-IoannaKato, HirohitoRyan, David K.Bold, Richard J.Kato, KenRybak, LeonardBolle, CordeliaKato, KoichiRychkov, GrigoriBollini, SvevaKato, KumikoRyckman, Kelli K.Bolmont, TristanKato, SouichiroRyoo, Hyun-MoBoltje, Thomas J.Kato, Takamitsu A.Sá, RosáliaBolukbasi, FatihKatoh, MasaruSaba, Nakhle S.Bombarda, IsabelleKatoh, ShigekiSacchetti, LuciaBombelli, PaoloKatsantonis, DimitriosSaccone, ValentinaBomben, RiccardoKatsila, TheodoraSachse, Frank B.Bomprezzi, RobertoKatsoris, PanagiotisSadakane, MasahiroBonacina, LuigiKatsuya, TomohiroSadik, RiadhBonaventure, PascalKattner, LarsSadkowski, TomaszBonfield, Tracey L.Katus, HugoSadovsky, YoelBonikowski, RadosławKauppinen, AnuSaeki, IkuyoBonventre, Joseph V.Kaur, SukhjiwanSaez, CarmenBoonkaew, BenjawanKaur, TejbeerSafa, AhmadBooth, BrianKawabata, SaneyukiSaferding, VictoriaBora-Singhal, NamrataKawada, KenjiSagi, AmirBorbély, YvesKawada, ManabuSaher, GesineBordignon, VilceuKawahara, EiSahin, ÖzgürBorghini, AndreaKawai, Vivian K.Sahlén, PelinBorkovich, Katherine A.Kawakami, SusumuSahrawy, MariamBormann, JörgKawamata, ShinSahrmann, PhilippBorole, Abhijeet P.Kawamura, AkifumiSahu, Saura C.Borovsky, DovKawamura, IzuruSaiano, FilippoBorràs, Francesc E.Kawamura, KazuhiroSaikumar, PothanaBorrás-Hidalgo, OrlandoKawano, KouichiroSainaghi, Pier PaoloBorroni, RiccardoKawaratani, HidetoSaini, SharanjotBorroto-Escuela, DasielKawasaki, IchiroSainlos, MatthieuBosch, ChristineKawashima, MakotoSaisho, YoshifumiBosco, DomenicoKawasumi, KohSaito, HidetsuguBose, DebojitKawauchi, AkihiroSaito, HiroshiBose, UtpalKayano, Shin-ichiSaito, YoshinoriBoselli, EmanueleKazlauskas, AndriusSaito, YoshiroBoskou, DimitriosKe, YouqiangSaitoh, AkiyoshiBotella, JimmyKeep, RichardSaitoh, MasaoBoth, SanneKeir, IainSajeva, MaurizioBottari, F.Kelleher, Fergal C.Sajic, MarijaBottino, Marco C.Kellenberger, StephanSakai, AtsushiBöttner, MartinaKeller, Amy CelesteSakakura, AkiraBouaïcha, NoureddineKeller, Evan T.Sakuma, KunihiroBoulter, LukeKeller, Jesse J.Sakurai, ManabuBouquillon, SandrineKelley, ThomasSakwe, AmosBourdon, Jean-ChristopheKellner, FranziskaSalas-de La Cruz, DavidBourgeois, DominiqueKellner, RonnySalazar, GloriaBoutinguiza, MohamedKelly, CliveSaldaña, GuillermoBove, Kevin E.Keltanen, TerhiSalek, RezaBowater, RichardKelts, JessicaSalgado, JosefaBowen, Richard A.Kemp, KevinSalhia, BodourBowling, HeatherKennedy, PatrickSalih, ErdjanBox, NeilKent, Robert M.Salim, VonnyBoyd, MarkKersten, SanderSalini, Michael J.Boyom, Fabrice FekamKeyaerts, MarleenSalinthone, S.Braathen, LasseKhadka, ManojSal-Man, NetaBradley, Walter G.Khaliulin, IgorSalomone, FedericoBraeuning, AlbertKhan, ImranSaltel, FrédéricBragado, M.J.Khan, MushfiquddinSalton, MaayanBrait, MarianaKhanna, NidhiSaluja, AshokBramanti, VincenzoKhazaie, KhashayarshaSalvati, BrunoBrambilla, EugenioKhoo, Bee LuanSalvato, FernandaBrambilla, VittoriaKhoobchandani, MenkaSalvatore, Ralph NicholasBranchini, AlessioKhozin-Goldberg, InnaSalvatore, SilviaBrand, LuiseKhuder, Sadik A.Samalin, E.Brás, NatérciaKhurshid, ZohaibSamant, Tanay S.Brastianos, Priscilla K.Kibriya, Muhammad G.Samarakoon, R.Braunstein, Evan M.Kiddle, GuySamuel, TemesgenBravo López, Susana B.Kidoaki, SatoruSamuels, Mark E.Bravo, JeronimoKiesslich, TobiasSan Martin, CarmenBreckenridge, RossKietzmann, ThomasSánchez Alcázar, JoséBrecker, LotharKihm, Lars P.Sanchez, David V.P.Breckpot, KarineKikuchi, EijiSanchez, Diego H.Brehme, MarcKim, ByeongilSanchez, GoarBreitenfeld Granadeiro, LuizaKim, ChaerinSánchez, SandraBrender, Jeffrey R.Kim, Gi JinSanchez-Dalmau, Bernardo F.Brennan, FrankKim, Gloria J.Sanchez-Garcia, IsidroBresson, JustineKim, HoonSánchez-Moreno, ManuelBrewer, GaryKim, Hye RyounSancho, RocioBrewin, NickKim, Hyun AhSangelaji, BahramBricknell, IanKim, Hyung SikSanjust, EnricoBridgewater, DarrenKim, Il-ManSanna, FabioBriedé, JaccoKim, Jae YoungSano, ShigetoshiBrier, Michael E.Kim, JinSantamaría, EstrellaBriles, David L.Kim, Jin MoonSantarelli, LoryBrinchmann, Monica F.Kim, Jin-BomSantella, LuigiaBrites, CarlaKim, Jin-WookSanter, Frédéric R.Briza, PeterKim, Jong BumSantini, ValeriaBroadbent, AndrewKim, JoungmokSantino, AngeloBrochier, CamilleKim, JulianSanto, Carmela DeBroderick, LoriKim, Ki-HongSantoni, MatteoBroderick, Tom L.Kim, Kyung-MinSantos, Ana PaulaBrona, MurphyKim, Kyung-SuSantos, Tírcia C.Bronisz, AgnieszkaKim, Na-HyungSantos-Gallego, CarlosBrooks, Amanda E.Kim, Sang-WeSantra, Dipak K.Brooks, AnnaKim, Seok JinSantulli, GaetanoBrossaud, JulieKim, Soo-UnSarath, GautamBrough, DavidKim, SukSarkar, ChinmoyBrough, LouiseKim, Sung-HoonSarker, DipakBroussard, Josiane L.Kim, Sung-WhanSarnyai, Z.Brown, GeoffreyKim, Tae IlSasaki, MasanoriBrown, JamesKim, Won HoSasaki, SeiBrown, LewisKim, Woo-JaeSasazuki, ShizukaBrown, Neil AndrewKim, Yong SooSato, MasahiroBruce, Erica D.Kim, Yong-JuneSato, MinoruBruining, NicoKim, YoungsooSato, Trey K.Brummelte, SusanneKim, Yun KyungSato, TsutomuBrun, NicolasKim, Yun-BaeSato, YuichiroBrundo, Maria ViolettaKimura, HidetoSatoh, ShigeruBrunelleschi, SandraKimura, IkukoSato-Nara, KumiBrunetti, CeciliaKimura, TadashiSator-Katzenschlager, SabineBrunker, KirstynKimura, TomokiSatou, RyousukeBrunner, GerdKing, Bonnie L.Sattar, SampurnaBrusselaers, NeleKing, Mary LouSattler, RitaBrustmann, HermannKing, MikeSaura, MaríaBryson-Richardson, RobertKinghorn, Kerri J.Saux, OlivierBu, XianheKinnula, HannaSavage, David B.Bubici, GiovanniKinoshita, TakayoshiSavaraj, NiramolBuccheri, MarinaKipp, Anna PatriciaSaville, RobertBuckley, M.Kirby, RalphSavoia, DianellaBucolo, ClaudioKirsch, ThorstenSavopoulos, ChristosBudak, HikmetKirschning, Carsten J.Sawada, HiroshiBuechler, ChristaKishimoto, YoshimiSawada, KenjiroBuenzli, Pascal R.Kishimoto-Yamada, KeikoSawai, HirofumiBugg, Timothy D.H.Kisiel, John B.Saxena, PraveenBühling, FrankKissling, JonathanSayers, Thomas J.Buján, NoemíKitamura, NaokiScafoglio, ClaudioBullock, AnthonyKitatani, KazuyukiScalise, MariafrancescaBullon, PedroKitchen, Scott G.Scanes, Colin G.Bunimovich, Yuri L.Kittner, Jens M.Scarlata, SuzanneBurghardt, RobertKiyama, RyoitiSchachter, MichaelBurke, MarkKiyan, YuliaSchaefer, JeremyBurse, AntjeKleeff, JörgSchaefer, MichaelBus, James S.Klegeris, AndisSchaffler, Mitchell B.Busardo, FrancescoKlein, KerstinSchäffner, AntonBuscarinu, Maria ChiaraKleinekathöfer, UlrichSchalken, JackBusch, HaukeKleuser, BurkhardSchatten, HeideBushley, KathrynKlimyuk, VictorSchausberger, PeterBustamante, AlejandroKlussmann, EnnoScheffler, Tracy L.Butlin, MarkKluza, JéromeScheiner, StefanCaballero, JulioKnapp, DeborahScheinman, RobertCabanillas, BeatrizKnapp, StefanScheper, VerenaCabarcos, PamelaKnecht, HansScherer, GüntherCabral, HoracioKnight, ChristopherSchiefer, Isaac T.Cabrera-Fuentes, HectorKnight, Jason S.Schierwater, BerndCaceres, AlejandroKniss, Douglas A.Schiffmann, RaphaelCaffarel, María MuñozKnock, Greg A.Schindler, ThomasCaffarelli, CarloKnott, RachelSchinke, ThorstenCahalon, LioraKnowles, Margaret A.Schirmer, BastianCahill, Abigail E.Kobayashi, HiroshiSchjoldager, Katrine T.-B.G.Cahoon, Aubrey BruceKobayashi, SatoruSchlaepfer, Isabel R.Cai, LuKobayashi, TatsuyaSchmid, MarkusCairncross, CarolynKobeissy, Firas H.Schmidt, JochenCairo, GaetanoKobori, HiroyukiSchmidt, MonicaCairrão, ElisaKocarnik, Jonathan M.Schmidt-Arras, DirkCalado, ÂngeloKöcher, ThomasSchmitt, JoachimCalaf, Gloria M.Kocmarek, Andrea L.Schmittgen, TomCalcagno, A.Koczera, PatrickSchmitz, GerdCalcutt, MichaelKodama, DaisukeSchmitz-Linneweber, C.Calì, TitoKoduru, Janardhan ReddySchnackenberg, LauraCalokerinos, AntonyKoea, JonathanSchneider, BarbaraCalorini, LidoKoga, FumitakaSchneider, BerndCalvisi, Diego F.Kogel, Karl-HeinzSchneider, DavidCalzavara-Pinton, P.Koh, HyongjongSchneider, JochenCalzetta, LuiginoKoh, TimothySchneider, RaphaëlCamarda, KyleKohen, RonSchoenhagen, PaulCambier, SébastienKohno, Jun-YaScholz, GlenCameli, NormaKohri, MichinariScholz, SonjaCampanale, Joseph P.Koistinen, HannuSchonthal, AxelCampanella, MichelangeloKojima, ChieSchoonheim, Menno M.Campanero-Rhodes, M.A.Kojima, MasamiSchrag, MatthewCampbell, ChristopherKojima-Yuasa, AkikoSchraufstatter, Ingrid U.Campbell, LeeKojo, ShosukeSchrauwen-Hinderling, V.B.Campbell, MichaelKokai, Lauren E.Schrempf, AlexandraCampello, SilviaKolasinski, Kurt W.Schrittwieser, StefanCampione, MarinaKolassa, Iris-Tatjana KolassaSchroeder, DanaCamporeale, AnnalisaKoller, MartinSchroeder, GrzegorzCampos Rosa, JoaquínKollmar, OttoSchroeder, HilkeCañada, F. JavierKomatsu, DavidSchroeter, MichaelCanini, AntonellaKomatsu, ShuheiSchulenburg, HinrichCañizares, CarmenKomis, GeorgeSchulz, Benjamin L.Cannavo, EldaKomohara, YoshihiroSchulz, Wolfgang A.Cao, LingKomoike, YutaSchwartz, GaryCaparrotta, LauraKondo, EisakuSchwarz, Steven M.Capel, FrédéricKondo, HidehiroSchweiger, Michal R.Capitanio, NazzarenoKondo, ToshihiroSchwiebs, AnjaCapodaglio, AndreaKong, Won-SikSciarretta, SebastianoCappelletti, VeraKonig, HeikoScicchitano, PietroCapperucci, AntonellaKoninckx, Philippe R.Scorziello, AntonellaCaprari, PatriziaKonishi, HirotakaScott, DanielCaramella, CarlaKonkol, YvonneScott, NaomiCarapelli, AntonioKonno, KatsuhiroScotti, LucianaCarattino, M.D.Kono, HiroshiScovassi, Anna IvanaCarbajo-Pescador, SaraKooijman, RonScozzafava, AndreaCarcillo, JosephKooiman, KlazinaScudiero, RosariaCardarelli, FrancescoKoon, Hon WaiSebastiani, FedericoCardin, ChristineKoonen, Debby P.Y.Seca, AnaCarlinfante, GabrieleKootala, SujitSeca, Ana M.L.Carlomagno, ChiaraKopecki, ZlatkoSedger, LisaCarnevale, GianlucaKopel, PavelSeegmiller, Adam C.Carol, PierreKoperek, OskarSeela, FrankCaroli, ManuelaKoppara, TobiasSeeliger, ClaudineCarpenter, Margaret A.Korbelik, MladenSeeling, Joni M.Carr, CarolynKornek, MiroslawSeeman, Mary V.Carr, John P.Korsching, EberhardSefat, FarshidCarranca, CorinaKosova, KlaraSehorn, MichaelCarraro, MauroKostakis, IoannisSehrawat, ArchanaCarrasco, PedroKostin, SawaSeib, PhilippCarregal-Romero, SusanaKota, SatyaSeifalian, Alexander M.Carreras, IsabelKotloski, RobertSeiliez, IbanCarrì, Maria TeresaKotsaki, AntigoneSeiquer, IsabelCarroll, RonanKotsikorou, EvangeliaSeki, NaohikoCarrubba, AlessandraKouadio, James HalbinSekine, KenTaroCarter, Wayne G.Kouretas, DimitriosSekino, MasashiCaruso-Nicoletti, ManuelaKourist, RobertSelim, SamehCarvajal, MicaelaKourkoumelis, NikolaosSella, LucaCarvalho, AnaKourkoutas, YiannisSellam, AdnaneCarvalho, LuísaKousoulas, Konstantin G.Sellayah, DyanCasas, Ana M.Kouvelis, Vassili N.Sellner, JohannCasey, AlanKowalski, KonradSeltsam, AxelCash, PhillipKox, M.Selvamani, AmuthaCasini, GiovanniKoyama, YuSempere, LorenzoCaskey, MarinaKozlevcar, BojanSen, NilkanthaCasquero, Pedro A.Kozliak, Evguenii I.Sen, Stephanie E.Castaño, OscarKrackhardt, AngelaSena, Dayse F.Casteel, Darren E.Kramer, PhillipSenga, TakeshiCastell, AlinaKratochwil, ClaudiusSenneville, ÉricCastellano, OrlandoKratochwil, ClemensSenpuku, HidenobuCastellanos-Garzón, José A.Krautwald, StefanSeo, Hak SooCastiglioni, BiancaKrawczyk, PawelSerafini, GianlucaCastillo, PabloKrebs, MichaelSerafini, NicolasCastñeiras, AlfonsoKrepkiy, Dmitriy V.Sergeant, KjellCaswell, Clayton C.Kretz, MarkusSeroussi, EyalCataldi, T.R.I.Kreuzwieser, JuergenSerra, FranciscaCatanzano, OvidioKrishnaswamy, GuhaSerrano, MariaCatto-Smith, AnthonyKristian, TiborServillo, GiuseppeCatts, VibekeKrjutškov, KaarelSestili, SaraCauffiez, ChristelleKroncke, Brett M.Sethi, GautamCauli, OmarKrüger, MarcusSetyawati, Magdiel InggridCavalla, P.Kruger, Maria J.Seva, CatherineCavalli, RobertaKruyt, Frank A.E.Sgadò, PaolaCaviglia, Gian PaoloKrysinska, KarolinaSha, ZheCeasar, S.A.Krzystek-Korpacka, M.Shafirstein, GalCeccarelli, SaraKu, Seung-YupShah, DilipCehofski, LasseKuan, Yu-HsiangShah, NilayCelli, AnnaKuang, HuihuiShaikh, Mohd. FarooqCellinese, NicoKubota, KeiichiShaio, Young-JiCerchia, LauraKubota, NaotoShankar, NeelaabhCeriotti, AldoKucerik, JiriShanmugam, MuruganandanCeroni, DimitriKucukkal, TugbaShao, KanCeroni, FabiolaKukreja, SubhashSharma, AmrishCerretani, LorenzoKulak, NoraSharma, AtulCervantes, Jorge L.Kumada, TakayukiSharma, BheshCesare Marincola, FlaminiaKumar, GauravShaul, Yoav D.Cesselli, DanielaKumar, SandeepShavrukov, YuriChaban, ChristinaKumar, SathishShear, Neil H.Chae, Han-JungKumar, SushilSheikh, NadeemChai, Han-HaKumar, UjendraShemanko, CarrieChaidos, AristeidisKumazawa, YoshinoriShemfe, MobolajiChakrabarti, RatnaKundu, AishwaryaShen, HaihongChakraborty, AnirbanKunnimalaiyaan, M.Shen, LeslieChallacombe, Jean F.Kunz, WolframShen, Tang-LongChallet, EtienneKunze, GotthardShen, YangChamcheu, Jean ChristopherKuo, Hsing-ChunShenderovich, Ilya G.Chami, BelalKuo, Ping-ChungSherbet, G.V.Chan, Aden K.Y.Kuo, Tzong-FuSherer, NathanChan, DannyKuo, Ya-HueiSheridan, PaulChan, King MingKupfer, AlexanderSherman, Michael P.Chan, Kun-MingKuppe, ChristophSherrard, RachelChan, ThomasKurdowska, Anna K.Shiau, Chyuan-YuanChand, SourabhKurepin, Leonid V.Shieh, Tzong-MingChandaka, Giri KumarKurpisz, MaciejShieh, Wann-YunChang, ChengKurschus, Florian C.Shikata, MasahitoChang, Chia M.Kurtcuoglu, VartanShil, SuranjanChang, Chien-ChungKurz, TinoShim, Jung OkChang, Chih LongKusakabe, MakotoShim, Yhong-HeeChang, Chin-ChyuanKusumbe, AnjaliShimizu, IppeiChang, Ching-JinKuwahara, KeisukeShimizu, ShigeomiChang, Chin-SungKuwata, Keith T.Shimizu, ToshiyukiChang, Chun-CheKwa, Faith A.A.Shimobayashi, MitsuguChang, Fang-RongKwakowsky, AndreaShimoda, HiroshiChang, Hen-HongKweon, Oh-KyeongShimosawa, TatsuoChang, Hsin-IKwok, JessicaShin, Chan YoungChang, Ing-FengKwon, Tae GyunShin, Jae-HoChang, Jin HoKwon, Tae-HwanShin, Kwang-SooChang, JungshanKwon, Young-WanShinohara, Russell TakeshiChang, Ko-TungKwong, JosephShintani, YasushiChang, Lin-LiKyprianou, NatashaShiota, MasakazuChang, Long-SenKyzas, GeorgeShiozawa, YusukeChang, Shun-FuLaboisse, Christian L.Shipley, JanetChang, Te-ShengLachman, JaromirShiraishi, TakehikoChang, Wei-ChiaoLachowicz, Joanna IzabelaShirakami, YoheiChang, Wen-ChiLafontaine, Denis L.J.Shirasawa, KentaChang, Wen-WeiLaforenza, UmbertoShirure, VenkteshChao, ThomasLahiri, AmitabhaShityakov, SergeyChapel, AlainLahooti, HooshangShivanna, BinoyCharchar, FadiLai, Christopher W.K.Shivapurkar, NarayanCharlton, ClivelLai, Hung-ChengShoenfeld, YehudaChassaing, BenoitLai, Hung-MingShoham, MenachemChater, CasparLai, Shih-WeiShoji, KensukeChatterjee, JhinukLaity, Peter R.Sholkamy, EmanChatzizisis, Yiannis S.Lam, Kim-HungShopsowitz, KevinChaudhry, ParveshLamas, José RamónShoshi, KikuchiaChauhan, ArunLamb, DavidShrestha, Lok KumarChaves-Pozo, ElenaLamers, Susanna L.Shrivastava, AbhishekChelland Campbell, SaraLan, LanShu, Mi ChungChen, BanglinLandete, José MariaShumilov, AnatoliyChen, Chao-JungLandi, AbdolamirSicher, Richard C.Chen, Cheng-LungLandriscina, MatteoSiddiquee, Tasneem A.Chen, ChiLane, Hsien-YuanSiddiqui, Rafat A.Chen, Chih-ChengLanekoff, IngelaSidi, YechezkelChen, Chiing-ChangLange, Kathrin M.Sidorenko, Viktoriya S.Chen, ChongyiLangenhan, TobiasSidransky, EllenChen, Chung-YiLansdon, Eric B.Siebel, Christian W.Chen, Chun-JungLao, T.T.Sieber, FritzChen, HuaiqiongLaplante, MathieuSieber, JonasChen, HuapingLaPres, John J.Siebuhr, A.S.Chen, Jiann-ChuLaranjo, MartaSiegfried, KelleeChen, JiaoLarena, InmaculadaSieiro, CarmenChen, Jih-JungLaroche, Céline LarocheSier, CornelisChen, KevinLarsen, FlemmingSiewert, BiankaChen, LiLarsen, Paul B.Sigala, SandraChen, Miaw-LingLarson, Nicholas B.Sikkema-Raddatz, BirgitChen, Po-ChiaLarsson, ChristerSilbert, BenjaminChen, QiLaSalle, JanineSilikas, NickChen, Qi-YinLash, LawrenceSill, HeinzChen, TianbaoLassmann, HansSillence, DanChen, Tzong-YuehLassot, IrénaSilljé, Herman H.W.Chen, XingLatinovic, OlgaSilva, Jorge CarvalhoChen, Y.-M.Lattanzi, WandaSilva, Luís R.Chen, Yei-TsungLau, Kin-Hing WilliamSilva, SnChen, Yen-HaoLau-Cam, Cesar A.Silverberg, Donald S.Chen, Yi-HungLaunikonis, BradleySimecka, Jerry W.Chen, YuLaurenceau, EmmanuelleSimeon, VittorioChen, Yu-ChingLaurienzo, PaolaSimeone, KristinaChen, YuhangLauzon, Carol R.Simirgiotis, MarioChen, Yung-ChangLavandera, IvanSimon, Scott I.Chen, Yun-WenLaville, MauriceSimons, Sinno H.P.Chen, Zhe-ShengLavrik, Inna N.Simons-Burnett, AndreanChen, ZhihangLawlor, KateSimpson, CraigChen, ZhongLaxton, Ross C.Simpson, JohnCheng, Fong-YuLay, Jackson O.Simpson, M.R.Cheng, Heung-ChinLazarević, VladimirSims, Ian M.Cheng, Jing-JyLazartigues, EricSingh, AnilCheng, Juei-TangLazzarato, LorettaSingh, AshutoshCheng, Kuang-HungLazzeri, ChiaraSingh, Atul KumarCheng, S.S.Le Hir, RozennSingh, Chandra K.Cheng, Ting-Yuan DavidLe Questel, Jean-YvesSingh, JagdishCheng, YongLe, MinhSingh, KaranCheon, Yong-PilLeach, KatieSingh, KashmirCheong, Yong HwaLeanza, LuigiSingh, Pramod K.Cherbuy, ClaireLeask, AndrewSingh, PrashantCheriyath, VenugopalanLebaron, RichardSingh, RajanChermette, HenryLebaron, SimonSingh, Reetu R.Chern, Ming-KaiLebeck, J.Singh, SeemaChevali, VenkataLebleu, BernardSingh, SuveerChevallier, NathalieLebre, Maria C.Sinha, AmitChew, Li-JinLechel, AndreSinnett, DanielCheynier, VéroniqueLechpammer, MirnaSipes, NishaChhatbar, PratikLecureur, ValerieSips, Patrick Y.Chiang, Hsiu-MeiLedesma, Maria DoloresSiracusa, LauraChiang, Meng-TsanLedoux, MarkSiristatidis, CharalamposChiang, Tai-AnLee, Bee WahSisodia, Sangram S.Chiang, Yu-ChungLee, Byung C.Sita, GiuliaChiba, YoshihikoLee, Che-HsinSiu, ParcoChien, Hung-YuLee, ChiSiwek, AgataChiesa, MarcoLee, Chia-HungŠkalko-Basnet, NatašaChiessi, EsterLee, Dae HoSkendros, PanagiotisChilds, Gwendolyn VaughnLee, Dong HunSkinner, JonathanChilton, RobertLee, GabsangSkot, LeifChim, StephenLee, Gwo-Shu MarySkuli, NicolasChimenti, SergioLee, Hsiang-ChiehSkundric, Dusanka S.Chin, MichaelLee, Hsin-ChenSkwarczynski, MariuszChing, Wei-MeiLee, HueiSleutel, MikeChinnam, Parameswara RaoLee, Huei-JaneSleutels, TomChiou, Chun-TangLee, Hye SukSłoczyńska, KarolinaChirumbolo, SalvatoreLee, J.-H.Slominski, AndrzejChisholm, John D.Lee, Jae-HoSlovin, Janet P.Chitarra, WalterLee, JandeeSlusarenko, Alan J.Chiu, Ing-MingLee, Jen-AiSmagula, Stephen F.Chiu, Ka Fung PeterLee, JiyounSmani, YounesChiurazzi, MaurizoLee, Juliana Tsz YanSmiesko, MartinChiurchiù, ValerioLee, Jun SikSmirnovas, VytautasChiva-Blanch, GemmaLee, Keun-HyeungSmit, Jasper V.Cho, Hyun-JeongLee, Kin Wah TerenceSmith, Cody J.Cho, Ssang-GooLee, Kuo-KauSmith, DarrinChobot, VladimirLee, Ming-FenSmith, Kylie J.Choi, Eun-KyoungLee, Moo-SeungSmith, MichaelChoi, Eun-MiLee, Myung KooSmith, MicholasChoi, Hyung-KyoonLee, Sam W.Smith, RachelChoi, Jang HyunLee, Sang-HanSmith, Tim A.D.Choi, Jor-ShanLee, SanghyeobSmits, AnthalChoi, WoonyoungLee, SeoulSmolenski, GrantChoi, Yoo SeongLee, Tzong-ShyuanSmolock, Christopher J.Choi, Yung HyunLee, Vincent WsSmýkal, PetrChomilier, JacquesLee, Y.-H.Smyrniotis, VassiliosChong, Parkson Lee-GauLee, Yi-JangSmyth, Lesley A.Chopp, MichaelLefèvre, ThierrySoares, HelenaChorianopoulos, Nikos G.Leffler, JonatanSobczak-Thépot, JoëlleChou, AngelaLehmann, ChristianSobin, CasChou, Kuo-ChenLehto, Vesa-PekkaSobolewski, MarissaChou, Wing-MingLei, BenfangSoggiu, AlessioChoudhury, MalayLeigh, Spencer A.Sohl, ChristalChougule, MahavirLeitão, AnaSohn, KaiChowdhury, KamalLeite, ClaudiaSohrabji, FaridaChren, Mary MargaretLemieux, M. JoanneSokolova, ViktoriyaChristiaens, OlivierLenaerts, KaatjeSolà Alberich, RosaChristofidou-Solomidou, M.Lenaz, GiorgioSolano, FranciscoChrzanowski, ŁukaszLeng, RogerSolecka, JolantaChu, Chun-HungLennox, KimSoliman, Khairy M.Chu, DafengLentini, GiovanniSolinís, M. ÁngelesChu, ShidongLenucci, Marcello SSoltani, AliChua, MelvinLeón, GerardoSomasundaram, R.Chuang, Chin-KaiLeonard, StephenSomers, DavidChuang, Lea-YeaLeong, David TaiSommerer, NicolasChuang, Yung-JenLeonhard, MatthiasSon, Chang-GueChudasama, VijayLeonhardt, Ralf M.Son, Deok-SooChueh, AnderlyLeoni, AlbertoSon, Mi-WonChun, Byung-SooLeopold, Jane A.Song, GuoqingChun, Kyung-SooLephart, Edwin D.Song, Jia L.Chung, ChuhanLeporatti, StefanoSong, Young ChaeChung, Eun JooLeppänen, JenniSong, YuyuChung, Yong-AnLessard, ChristopherSonkoly, EniköChyau, Charng-CherngLesueur, DidierSonnenberg, Anton S.M.Ciaccio, MarcelloLeung, Chung-HangSonnweber, ThomasCiaudo, ConstanceLeung, Elaine Lai-HanSonpavde, GuruCiccacci, CinziaLeung, Ricky Yuet-KinSonsalla, Patricia K.Ciccozzi, MassimoLeuridan, ElkeSorce, SilviaCicero, ArrigoLevänen, BettinaSoreq, HermonaCichero, ElenaLevato, RiccardoSoriano, ElenaCignetti, AlessandroLevi, MosheSorrentino, ElenaCilia, MichelleLevine, Andrew J.Sosa, SilvioCimino, FilibertoLevingstone, TanyaSosnowska, AnitaCindrova-Davies, TerezaLevraud, Jean-PierreSotome, ShinichiCiogli, AlessiaLevy, David N.Sottnik, JosephCipolla, DavidLevy, MichaelSoucy, ShannonCirillo, GiuseppeLevy, StevenSouders, Colby A.Cisowsky, JaroslawLewis, James H.Souidi, MaamarCitro, ValentinaLewis, RandolphSoulier, JeanCitterio, DavideLi, DongmeiSoutham, Andrew D.Ciulu, MarcoLi, FuSoveral, GraçaClark, Lindsay V.Li, GuoqiangSpanakis, MariosClaus, HaraldLi, Hsing-HuiSpandou, EvangeliaClausen, Rasmus PrætoriusLi, JiannengSpanò, StefaniaClawson, Gary A.Li, JunSparkes, JamesClem, Brian F.Li, Jun-XuSpeck, Peter G.Cleveland, Beth M.Li, Kuo-BinSpee, BartClevenger, Charles V.Li, LeiSpence, TaraClowry, Gavin J.Li, LiSpendier, KathrinCoan, P.M.Li, LingSpickett, Corinne M.Coates, Christopher J.Li, LinlinSpingler, BernhardCoccheri, SergioLi, NingSpiotto, Michael T.Cocetta, GiacomoLi, QiangSpizzo, GilbertCochran, BlakeLi, TiangangSpormann, Alfred M.Coco, SilverioLi, WanlongSprenger, HeikeCodacci-Pisanelli, GiovanniLi, XiaoliSpruck, CharlesCoelho, José A.Li, YangSrebnik, SimchaCognasse, FabriceLi, YangmingSreedasyam, AvinashCohen, DanLi, YefuSridharan, VijayalakshmiCohrs, RandallLi, Ying-JiSrinivas, KeerthiCojocaru, VladLi, YonggangSrivastava, AkhilCole, John W.Liang, RongguangSrivastava, RakeshColeman, William B.Liang, Xue-HaiSrivatsan, Eri S.Collavin, LicioLiao, Francesca-FangSrivenugopal, KalkunteCollawn, Sherry S.Liao, Jen-ChungStaal, JensCollin, MatthewLiao, LiliStanika, RuslanCollin, Rob W.J.Liao, You-DiStanley, JoneColloc’h, NathalieLiapi, CharisStanta, GiorgioCollongues, NicolasLibbrecht, RomainStanton, Robert C.Colombo, Gualtiero I.Liebman, Joel F.Staple, AlanColson, NatalieLiebrand, Thomas W.H.Stec, JozefColvin, RobertLiechti, Matthias E.Steen, Ida HeleneComai, StefanoLiew, Woei ChangStefanidou, Maria E.Conant, KatherineLightfoot, J. TimothyStefano, CassanelliConboy, JohnLigterink, WilcoStefano, CescoConigliaro, AliceLiguori, C.Stehle, Jörg H.Conlan, SeanLiguori, MariaSteiner, Joseph P.Connelly, Kim A.Lim, BoraSteinhilber, DieterConner, MatthewLim, Do-SunStelinski, LucaszContassot, EmmanuelLim, EdwinStephenson, Sally-AnneConte, EnricoLim, Eun-KyungSteup, MartinConte, SasiLim, Ki-ByungStevanato, PiergiorgioCook, Atlanta G.Lim, Kyung-MinSteven, SebastianCook-Mills, Joan M.Lim, Sung-JigStevens, Richard G.Cooney, AustinLimon, AgenorSteves, Dr. Claire JoanneCooney, RalphLin, Cheng-WenStewart, AnthonyCooper, ArthurLin, Chi-ChenStifani, StefanoCooper, Timothy K.Lin, Chiou-FengStillaert, FilipCooper, William T.Lin, GuitingStilli, DonatellaCopple, IanLin, HoStine, Keith J.Corbett, Harriet J.Lin, HuiStocco, CarlosCorbetta, SabrinaLin, Hung WenStock, ChristianCorcoran, Brendan M.Lin, Hung-YinStocker, RetoCordat, EmmanuelleLin, Kwang-HueiStoelzel, FriedrichCordero, PaulLin, LiStone, James R.Cornelissen, BartLin, Liang-InStone, KariCorniola, RikkiLin, Liang-TzungStope, Matthias B.Correia, SofiaLin, Li-HueiStopsack, Konrad H.Correia-de-Sá, PauloLin, Ming-KuemStorer, Nicholas P.Correia-Neves, MargaridaLin, Pao-HwaStork, Christian J.Corrigan, FrancesLin, Pei-HuiStrano, SabrinaCorsetto, Paola A.Lin, Tsan-PiaoStraub, Rainer H.Cortese, FrancescaLin, Yuan-ChungStrauss, SarahCortese, KatiaLindenmann, JörgStrekalova, TatyanaĆosić, JasenkaLindsay, Laura A.Strekowski, LucjanCosset, François-LoïcLindstrand, AnnaStreng, AndreaCosta, Catalina MartinezLinert, WolfgangStrick, ReinerCosta, FabrizioLing, HuiStrickland, Dudley K.Costa, José LuisLing, Maurice HtStritzker, JochenCosta, Paulo J.Lingappan, KrithikaStrollo, FeliceCosta, RuiLinnstaedt, SarahStuart, JeffCostagliola, CiroLio, DomenicoStudzinski, George P.Costantino, LucaLiou, Ying-MingStuppia, LiborioCôté, Jean-FrançoisLipke, Peter N.Stura, Enrico A.Coto, EliecerLippert, EricStyrsky, John D.Coulter, Jonathan A.Lips, Katrin S.Su, BaofengCoupland, Lindsay J.Liu, Alvin Y.Su, ChunmingCourtney, Brian K.Liu, BinSu, Pi-GueyCourty, Pierre-EmmanuelLiu, BotaoSu, Ruey-ChyiCourville, Elizabeth L.Liu, C.Y.Su, TingCousin, XavierLiu, Chao-LinSu, Wen-TaCouttet, PhilippeLiu, DelongSuades, JoanCoutts, RobertLiu, DongxuSubramaniam, PrasadCoyle, BethLiu, FanSucharov, CarmenCraig, Andrew W.B.Liu, FengSudhir, Putty-ReddyCraig, Timothy P.Liu, JianSugimura, HaruhikoCrava, Cristina M.Liu, Jinn-LiangSukumari-Ramesh, SangeethaCray, James J.Liu, JinxuanSulek, KarolinaCrespi, MartinLiu, Jun-JenSullards, CameronCrisafulli, ConcettaLiu, LiSumitani, Jun-ichiCristiani, PierangelaLiu, QiSun, DaekyuCroce, Anna CletaLiu, QingSun, FenCroset, MartineLiu, RengyunSun, JieCross, Carroll E.Liu, ShengquanSun, MingCrotti, TaniaLiu, Shih-AnSun, MingkuanCrowe, David R.Liu, Shing-HwaSun, QiCuesta-Seijo, Jose A.Liu, ZhixiaSun, WenyueCui, HaitaoLiu, ZiqingSun, YingCui, JuanLizee, Gregory A.Sun, YingjieCulot, MaximeLleò, Maria M.Sun, ZhifuCulotta, Valeria C.Lo Muzio, LorenzoSundar, Isaac KirubakaranCummings, BrianLo, Hui-ChenSundd, PrithuCunningham, PatrickLo, Shih-YenSung, PatrickCurtin, ChrisLocatelli, MarcelloSuperti, FabianaCurtis, WayneLoebel, AntonySupuran, Claudiu T.Czosnek, HenrykLofrumento, Dario D.Surber, ChristianCzosnyka, MarekLoizides-Mangold, UrsulaSurguchov, AndreiD’Anna, FrancescaLokshin, AnnaSussan, ThomasD’Souza-Schorey, CrislynLombardi, PaoloSussman, Mark A.Da Cruz-Höfling, Maria AliceLonardo, AmedeoSutherland, AndrewDa Silva, A. MoreiraLoo, Jennifer D.Sutton, Timothy A.D’Abusco, Anna ScottoLood, ChristianSuzuki, HiroetsuDacher, MatthieuLoos, KatjaSuzuki, Hiroshi I.Dacheux, Jean-LouisLópez-Guerrero, AntonioSuzuki, NobuhiroDaftarian, PirouzLopez-Llorca, LuisSuzuki, TakuyaDag, ArnonLophatananon, ArtitayaSuzuki, Yuichiro J.Dagnino, LinaLoquet, AntoineSvenja, HardtkeDague, EtienneLora-Tamayo, JaimeSvensson, Katrin J.Dahaba, Ashraf A.Lorberboum-Galski, HayaSwadźba-Kwaśny, M.Dai, ShaojunLord, JanetSwales, CatherineDai, Shu-MeiLord, MeganSwamidass, S. JoshuaDaidone, Maria GraziaLorenz, KristinaSwarnkar, GauravDailey, HarryLorenzini, AntonelloSwarup, VimalDailianis, StefanosLorenzo, ÓscarSweazea, KarenDall’Acqua, StefanoLorico, AurelioSwerdlow, RussellDall’Olio, FabioLou, Tsung-LinSwevers, LucDamaraju, SambasivaraoLoudig, Olivier D.Switonski, MarekDamia, GiovannaLove-Gregory, Latisha D.Sylla, MaiteDamico, MicheleLoveridge, JoelSylvester, Paul W.Da’na, EnshirahLowe, RohanSylvie, MonferranDanan, GabyLoxham, MatthewSzasz, TheodoraDandekar, ThomasLu, Chi-YuSzczerba, WojciechDane, FennyLu, HuaSzekely, GyorgyD’Angelo, LiviaLu, LinchaoSzeto, Yim-TongD’Angiolella, VincenzoLu, Mei-ChinSzyszkowicz, MieczysławDanielson, Kirstie K.Lu, Ming-WeiTaboureau, OlivierDanielson, NeilLu, PaulTabuchi, YoshiakiDanila, Daniel C.Lu, Pei-JungTacer, Klementina FonDanilenko, MichaelLu, ShenzhouTada, HayatoDarby, Ian A.Lu, ShuoTafesse, Fikadu GetaDare, AndrewLu, YangTagliabue, EldaDare, RyanLu, Yen-ShenTaguchi, YoshihiroD’Argenio, GiuseppeLu, YongkeTaheri, ArashD’Argenio, ValeriaLucacchini, AntonioTahriri, MohammadrezaDark, Michael J.Lucarini, GuendalinaTai, Helen H.Darland, TristanLuchetti, MIcheleTaira, JunseiDarling, SethLuco, Reini F.Tajmir-Riahi, H.A.Darvesh, SultanLuconi, MichaelaTakabe, KazuakiDarvin, MaximLudwig, ChristianTakagi, KiyoshiDas, AninditaLuetgehetmann, MarcTakahisa, KanekiyoDas, ArupLui, EdTakaki, AkinobuDasgupta, SantanuLui, Vincent Chi-HangTakamatsu, KazuhikoDashti, HassanLuigi, ScipioneTakano, KatsuraDavenport, AnthonyŁukasik, RafałTakanori, MarutaDavenport, MarkLukaszewski, AdamTakao, SonshinDaverey, AmitaLuna, Jonny E. DuqueTakatsuji, HiroshiDavey, Rachel A.Lung, Maria LiTakayama, YoshiharuDavidson, Nicholas O.Luo, HuachengTakayanagi, ToshiyukiDavies, KeithLuongo, LivioTakemori, HiroshiDavies, Kelvin J.A.Lupiáñez, José A.Takenaga, MitsukoDavies, KevinLupold, Shawn E.Takeshita, FumitakaDavies, LeoLuque, FranciscoTakeshita, HaruoDavies, MichaellŁuszczek-Trojnar, EwaTakeuchi, YukoDavis, Gregory D.Lutfy, KabirullahTakumi, ShigeoDavis, MindyLütken, HenrikTakyar, SeyedtaghiDavolos, DomenicoLutz, JensTalbert, Joey N.De Aberasturi, D.J.Luu, Doan-TrungTalou, Julián RodriguezDe Andrés Cara, Damián F.Lymperopoulos, AnastasiosTam, Wilson W.S.De Berardis, DomenicoLynd, NathanielTamaki, HideyukiDe Bernonville, T.D.Lyng, HeidiTamalis, DimitriDe Biase, DarioLyons, Paul E.Tamaru, TeruyaDe Bortoli, MicheleLyons, RussellTambi, RichaDe Cáceres González, F.F.N.Ma, Dik-LungTamborindeguy, CeciliaDe Feo, VincenzoMa, GuojiaTambur, Anat R.De Francia, SilviaMa, QingTamma, GraziaDe Geest, BartMa, WeiTamori, AkihiroDe Gelder, LeenMa, XingzheTamura, Ken-ichiDe Giorgi, UgoMa, YanTan, Eng-KingDe Guzman Strong, CristinaMa, YiyiTan, YuqingDe Jong, Jill L.O.Ma, YongjieTanahashi, HiroshiDe Jonghe, KrisMaccallini, CristinaTanaka, HiroshiDe Koning, DirkMaccari, GiuseppeTanaka, NobuyukiDe Kort, Laetitia M.O.Maccari, RosannaTanaka, ShigeoDe Kretser, David MorritzMacias, Rocio I.R.Tanaka, TakashiDe La Cruz, José PedroMack, Andreas F.Tanaka, TomoakiDe La Herrán, RobertoMacMillan, FreyaTanaka, YoshiyukiDe La Hoz, AntonioMacPherson, Rebecca E.K.Tang, Bor LuenDe La Luz Cádiz Gurrea, M.Madesis, PanagiotisTang, DamuDe La Monte, SuzanneMadhu, RajeshTang, Simon Y.De La Torre, XavierMaekawa, ShinyaTang, XiaojiaDe Lago, EvaMagalhães, Júlia M.C.S.Tang, XinyanDe Lange, PieterMaggioni, DanielaTanigawa, TetsuyaDe Mattei, MonicaMaggioni, DanieleTanonaka, KouichiDe Morais, R.M.S.C.Magiatis, ProkopiosTansey, JohnDe Noronha, Carlos M.C.Magnaldo, ThierryTanzi, E.De Paolis, AngeloMagne, DavidTaoka, YousukeDe Rivero Vaccari, Juan PabloMahajan, SahilTapia, Anibal AlejandroDe Ron, AntonioMaher, PamelaTappia, Paramjit S.De Rosa, Maria CristinaMahgoub, AmarTarantino, GiovanniDe Rosa, VivianaMahimainathan, LeninTaratula, OlenaDe Visser, S.P.Mahoney, Sara E.Tarro, GiulioDe Vos, JohnMahony, Timothy JohnTasca, GiorgioDe Vos, RicMai, SabineTashima, KimihitoDe Vries, CarlieMaier, PatrickTashiro, HirotakaDe, KuntalMaillard, IvanTassone, PierfrancescoDearden, John C.Maioli, EmanuelaTastet, LionelDebode, FrédéricMajewski, MichałTata, Ada MariaDebray, François-GuillaumeMajumder, MousumiTatakis, Dimitris N.Decker, MichaelMäkelä, Miia R.Tate, Rothwelle J.Deeg, CorneliaMakino, TakeshiTatemoto, YujiDeevska, GerganaMakishima, MakotoTatzelt, JörgDegano, IlariaMalafa, MokengeTaube, JoeDegens, HansMalamud, DanielTauchi, TetsuzoDeive Herva, Francisco J.Malde, AlpeshTava, AldoDekaban, Gregory A.Malemud, CharlesTavazzi, BarbaraDekker, FrankMalheiro, RicardoTavío, María Del MarDekker, MarloesMalhotra, VivekTavornpanich, SarayaDel Álamo, M.M.R.Malinauskas, MangirdasTay, Chor YongDel Vescovo, ValerioMalkoc, VeysiTayebati, Seyed KhosrowDelagoutte, EmmanuelleMalli, RolandTaylor, Anna M.W.Delerue, FabienMallikaratchy, PrabodhikaTaylor, CarolineDelgado, María Jesús SantosMallipeddi, Prema LathaTaylor, GrahamDelhanty, Patric J.D.Malm, HeliTaylor, JaniceDelisle, HélèneMalter, JamesTaylor, Zachary S.Dell’Era, PatriziaManabe, IchiroTaymans, Jean-MarcDelporte, ChristineMandolesi, GeorgiaTchapla, AlainDelRosso, MarioManetti, MirkoTeeri, TeemuDemitrack, EliseManga, PrashielaTeets, Nicholas M.Demorrow, SharonMangiatordi, Giuseppe FeliceTegeder, IrmgardDeng, QingMann, Karen P.Tejero, JesúsDeng, XufangManninger, MartinTelugu, Bhanu Prakash V.L.Deng, YongManor, DannyTemeyer, Kevin B.Deng, YongmingManouchehrinia, AliTemmam, SarahDenizot, B.Manrique, CamilaTemussi, Piero A.Dennenmoser, StefanMansley, MoragTendulkar, KetkiDentino, Andrew R.Mantamadiotis, TheoTeng, Ru-JengDermauw, WannesMante, Francis K.Teng, ShaoleiDesBordes, CharlesManthey, John A.Tennessen, Jacob A.Deshayes, SébastienManuali, ElisabettaTerada, NaokiDesmaële, DidierManzardo, A.M.Teramoto, HidetoshiDesmet, ElineMao, ChuanbinTerashima, TomoyaDessy, ChantalMao, MengTerracciano, DanielaDevesa, V.Marabese, MirkoTerrier, OlivierDevy, JérômeMarasco, DanielaTerry, Randall G.Dhaenens, ClarisseMarcal, HelderTeschke, RolfDhanasekaran, Saravana M.Marcel, V.Testai, Fernando D.Dhar, AnimeshMarcelli, AugustoTetlow, IanDhaubhadel, SangeetaMarcelo, FilipaTevosian, Sergei G.Dhondt-Cordelier, SandrineMarchesan, SilviaThannhauser, Theodore W.Dhoot, Gurtej K.Marchetti, PhilippeTheiss, CarstenDi Benedetto, BarbaraMarchini, SergioThelin, EricDi Fazio, PietroMarcos, MercedesThemstrup, LotteDi Fiore, Maria MaddalenaMarcos, MiguelThevelein, JohanDi Giannantonio, M.Marcovici, G.Thèvenod, FrankDi Giovanni, SimoneMargalef, MariaThiery, DenisDi Liegro, ItaliaMargaria, PaoloThijssen, Victor L.Di Luccia, AldoMargaria, ValentinaThilmony, RogerDi Maio, ErnestoMargaritis, Lukas H.Thomas, MichelleDi Paolo, AntonelloMaria José, R.L.Thomas, T.J.Di Pietro, CinziaMarin, Jose J.G.Thompson, KimDi Primio, R.Marini, Juan C.Thomsen, HaukeDi Sansebastiano, Gian PietroMariniello, LoredanaThomson, DanielDi Stefano, AntonioMarino, JosephThorin-Trescases, NathalieDiapari, MarwanMarkopoulos, Anastasios K.Thorlacius-Ussing, OleDias, JulianaMarkopoulou, OlgaThounaojam, MenakaDiaz, IsabelMarmiroli, MartaThünemann, AndreasDiaz, Mayri A.Marmiroli, NelsonThyagarajan, BharatDíaz-Sala, CarmenMarondedze, ClaudiusTian, LiDiccianni, Mitchell B.Maroni, PaolaTian, YingfangDickert, FranzMaroon, JosephTickner, JenniferDickstein, RebeccaMaroteaux, LucTie, JeanneDidonna, AlessandroMarsano, FrancescoTihkonov, VladimirDiegelmann, RobertMarsik, PetrTijeira, MartaDieplinger, BenjaminMarston, Denise A.Tikkanen, RitvaDieterlen, Maja-TheresaMartens, John W.M.Tiley, LaurenceDievart, AnneMartí, SérgioTilley, MichaelDikalov, SergeyMartienssen, Robert A.Tilton, Ronald G.Dimova, ElitsaMartin, AlastairTimoshenko, Alexander V.Ding, BaoqingMartin, FinianTiribelli, ClaudioDingwall, Andrew K.Martin, JessicaTittiger, ClausDinh, Dzung H.Martin, Luc J.Tliba, OmarDinwiddie, Darrell L.Martin, SophieTo, Kin-YingDionisi, DavideMartín-Aragón, SagrarioTocci, GiulianoDionisio, GiuseppeMartinelli, PaolaToietta, GabrieleDiot, ChristianMartinez, ManuelToju, HirokazuDioum, Elhadji M.Martinez-Arguelles, D.B.Tokoro, ChiharuD’Ischia, MarcoMartínez-Balibrea, EvaTokuyama, ShogoDivi, Rao L.Martínez-Campa, CarlosToldo, StefanoDixon, David A.Martinez-Castelao, AlbertoTölli, HannaDjouadi, FatimaMartínez-Chantar, María LuzTolosa, EzequielDoan, Ngoc DucMartínez-Mayorga, KarinaTomasetti, CarmineDobens, Leonard L.Martínez-Rivas, J.M.Tomasetti, MarcoDoddapaneni, RaviMartinez-Sanchez, AidaTomassetti, AntonellaDoh, Kyung-OhMartini, ClaudiaTomaz, Cândida TeixeiraDolferus, RudyMartini, XavierTomitaka, AsahiDolga, AmaliaMartino, SabataTomoaki, SakamotoDolga, Amalia M.Martinotti, GiovanniTomoda, HiroshiDoll, KennethMartins, Joana T.Tomofuji, TakaakiDolo, VincenzaMaruf, Abdullah AlTomonaga, KeizoDombrowski, NinaMaruyama, KeiTomsic, JernejaDomingos, Pedro M.Maruyama, KyonoshinTondi, GianlucaDonato, RosarioMarverti, GaetanoTong, Hoang V.Dondelinger, YvesMarzban, HassanTong, IrisDong, LanfengMas, EricTong, JingouDonly, B. CameronMasella, RobertaTong, Yat-ChingDonnini, SandraMashima, RyuichiTopakas, EvangelosDonzelli, ElisabettaMasi, AntonioTormo, José RubénDonzelli, SabrinaMason, RebeccaTorras, J.Doonan, JamesMasotti, AndreaTorras, JoanDoran, MichaelMassie, CharlieTorre, JuanDorfman, DamianMasters, Colin L.Torrens, FranciscoDormond, OlivierMastrangelo, Anna M.Torres, Carlos F.Dorner, AMastrocola, RaffaellaTorres, JaumeDossena, MartaMasuda, KiyoshiTorres, M.I.Douros, AntoniosMasui, KentaTorres-Aleman, IgnacioDoyle, HesterMasuo, KazukoTorres-Russotto, DiegoDoyle, SiamsaMate, SebastianTortiglione, ClaudiaDragsted, LarsMathura, Venkatarajan S.Tortora, P.Drakoulis, NikolaosMatin, AbdulTorzewski, MichaelDraper, IsabelMatoba, NobuyukiTosoian, Jeffrey J.Dreij, KristianMatos, Ana RitaToth, Eric A.Drickamer, KurtMatsuda, HisashiTouyarot, KatiaDriscoll, James J.Matsufuji, HiroshiTrabace, LuigiaDrosten, MatthiasMatsugi, MasatoTraber, MaretDryga, AnatolyMatsugo, SeiichiTrabolsi, AliDu, DongliangMatsui, KenjiTralau, TewesDu, K.-L.Matsui, Mary S.Tramice, AnnabellaDuangthip, DuangpornMatsui-Yuasa, IsaoTramontano, DonatellaDuarte, Julio D.Matsumoto, Ken-ichiroTran, Nhan L.Dubey, AshishMatsumoto, YasuhikoTranbarger, TimothyDubey, RaminMatsuoka, DaisukeTraweger, AndreasDubielecka, Patrycja M.Matsuoka, MakotoTreesukosol, YadaDuBois, Debra C.Matsushita, TakehikoTreves, SusanDubrova, YuriMatsuura, BunzoTrevino, JoseDudás, JózsefMatsuura, KazunoriTrevisi, LuciaDudziak, DianaMatsuzaki, ToshiyukiTrif, MonicaDuennwald, Martin L.Matta, CsabaTrincone, AntonioDuerksen-Hughes, PenelopeMatta, Jamie L.Tringali, CorradoDufau, Maria L.Mattei, FabrizioTringali, CristinaDuhamel, JeanMatthaei, HannoTripathi, PrateekDuman-Scheel, MollyMatthews, BenTristram, StephenDumez, SylvainMattijssen, FritsTrivedi, DarshanDumon, StephanieMattner, JochenTrivellini, AliceDunietz, Barry D.Maugeri-Saccà, MarcelloTrognitz, FriederikeDunne, Eileen M.Maupin, C. MarkTrollmann, ReginaDuong, Hai M.Mauriello, GianluigiTrollope, AlexandraDupureur, Cynthia M.Mavrogonatou, EleniTrosko, JamesDurán, I.Mavromoustakos, ThomasTrovato, Francesca MariaDurazzo, AlessandraMay, PhilipTrovato, GuglielmoDurbec, PascaleMayhan, William G.Trzciński, KrzysztofDuressa, DechassaMaynard, Desmond J.Tsaftaris, AthanasiosDuszenko, MichaelMazzoleni, StefanoTsai, Fuu-JenDutcher, James D.McAuley, Julie L.Tsai, JamesDwivedi, ChandradharMcCall, KimberlyTsai, Keng-ChangDwyer-Nield, Lori D.McCallum, Jason L.Tsai, Kun-LingEaly, AlanMcCamant, DavidTsai, SientangEamens, AndyMcCarty, Nami McCartyTsai, Tsung-YuEasmon, JohnnyMcCaughan, G.W.Tschierlei, StefanieEbrahim, WeaamMcCoard, S.A.Tse, William K.F.Echevarría, MiriamMcConathy, JonathanTsitsilonis, Ourania E.Ecke, ThorstenMcCullough, AmandaTsoi, JamesEckel, JürgenMccully, Kilmer S.Tsuchiya, KosukeEconomou, Christina N.McDaid, HayleyTsuchiya, YuichiEdelman, Arthur M.McDaneld, Tara G.Tsuji, MasahiroEder, Iris E.Mcdonald, CourtneyTsuji, PetraEder, MatthiasMcDonald, KarenTsuji, ShojiEdgren, TomasMcDougall, GordonTsujimura, SeiyaEdin, Nina JeppesenMcElroy, KyleTsunoda, IkuoEduardo-Figueira, MariaMcFarlane, Heather E.Tsurkan, Mikhail V.Edwards, Jeffrey G.McGarrigle, Eoghan M.Tsutsui, ShigeyukiEe, Pui-Lai RachelMcGarry, Roisin C.Tsuyama, NaohiroEferl, RobertMcGhee, James D.Tuaillon, E.Efird, JimmyMcGrail, MauraTuchscherr, LorenaEgawa, GyoheiMcGrath, ScottTucker, StevenEgea, MarcosMcgrew, Michael J.Tuckermann, JanEggeling, LotharMcIntyre, Jenifer K.Tuda, MidoriEggert, JullieMcMahon, Lawrence P.Tuffaha, Sami H.Ehlting, ChristianMcMillin, MatthewTuffery-Giraud, SylvieEhret, Georg B.Mcmorrow, TaraTulkens, PaulEhrich, MarionMcMurray, FionaTumanov, Alexei V.Ehrnhoefer, DagmarMcPhee, JamieTuna, DenizEid, NabilMcsharry, CharlesTung, Tao-HsinEilertsen, Karl-ErikMeade, Mark E.Tunuguntla, HariEisenreich, AndreasMécheri, SalaheddineTuomikoski, PauliinaEitsuka, TakahiroMechler, AdamTuriel, MaurizioEkinci, Elif I.Medici, SerenellaTurk, Benjamin E.Ekuni, DaisukeMedin, Jeffrey A.Turner, HelenEl Benna, JamelMehaffy, CarolinaTurner, Neil A.El Desoky, Ehab S.Mehra, ReenaTurner, ReneeElahi, ShokrollahMehrabi, ArianebTurpin, SarahElbayoumi, TamerMei, NanTurunen, Mikko P.Eldeeb, MohamedMeier, CarolaTusell, LlibertatElez-Martinez, PedroMeijer, H.J.G.Tuttolomondo, AntoninoElhanafi, Ahmad OmarMeijón, MónicaTuyaerts, SandraEliseev, Roman A.Meldolesi, JacopoTyakht, Alexander V.El-kereamy, AshrafMelillo, Rosa MarinaTzang, Bor-ShowElkouby, Yaniv M.Mellon, IsabelTzeng, Nian-ShengEller, FredMelnik, Bodo C.Tziomalos, KonstantinosEllies, L.G.Memarzadeh, KavehTziotzios, ChristosEllinger, JörgMemon, M.A.Tzortzakis, NikolaosElliott, Paul R.Ménard-Moyon, CéciliaUchida, ShizukaEllis, JohnMenassa, RimaUchio, YujiEllsworth, Darrell L.Mende, SusannUeda, AkiraEl-Matbouli, MansourMendes-Ferreira, AlexandraUehara, TomoyaEl-Mowafy, OmarMéndez-Ardoy, AlejandroUeno, Shu-ichiElsea, Sarah H.Méndez-Sánchez, NahumUeta, IkuoEl-Seedi, Hesham R.Menendez Ramos, J.C.Uhr, Jonathan W.El-Soda, MohamedMenendez, DanielUlisse, SalvatoreEl-Sohemy, AhmedMenendez, EstherUlivi, PaolaEmile, Jean-FrançoisMeng, XiangbingUllmann, G. MatthiasEmmert, SteffenMenkhorst, EllenUlsh, BrantEn Henegouwen, P.V.B.Menu, ElineUmberger, Reba A.Engberg, GöranMenzies, Robert I.Umemori, JuzohEngel, AndrewMercader, JosepUno, TomohideEngelberg, DavidMercati, FrancescoUrata, HidehitoEngelberth, JurgenMercer, CarolUrrestarazu, JorgeEngelund, Morten B.Mercuri, Nicola BiagioUssar, SiegfriedEnglish, NiallMerkel, OlafUsui, SoichiroEngström, MariaMerlin, GérardUysal, UtkuEnguita, FranciscoMerry, Troy L.Uzawa, AkiyukiEppler, ElisabethMessaritakis, IppokratisVaidya, BhuvaneshwarEpstein, AlanMettler, LiselotteVaidyanathan, GanesanEquils, OzlemMetz, LuanneVaiman, DanielErdbruegger, UtaMetzger, Dennis W.Vainio, Eeva J.Eri, RajMetzinger, LaurentVainonen, JuliaEriksson, OskarMetzler, MarkusVajro, PietroEritja, RamonMetzler-Wilson, KristenValdes, Ana M.Ersvær, ElisabethMeulenberg, ElineValdez, GregorioEscames, GermaineMeuris, BartValenti, LucaEsguerra, Jonathan Lou S.Meyer, Barbara J.Valenti, PieraEspen, LucaMeyers, GregorValentinuzzi, FabioEsperança, José M.S.S.Mian, M.A. RoufValentovic, MonicaEspinosa-Diez, CristinaMiccadei, StefaniaValero, ManuelEsposito, CiroMichalek, KatarzynaValiante, VitoEsposito, DeboraMichela, CastagnaValle, AdamoEsposto, SoniaMichelhaugh, Sharon K.Valli, VeronicaEsrafilzadeh, DornaMichels, AaronVallières, LucEssani, KarimMichetti, FabrizioValpuesta, VictorianoEssers, Marieke A.G.Michiels, CarineVan Aerschot, ArthurEstelrich, JoanMichl, Thomas D.Van Bodegraven, Ad A.Estes, Jacob D.Michos, A.Van Brabant Smith, AnjaEsteves, Ana CristinaMico, Juan AntonioVan Coppenolle, FabienEstevez, AnaMiddelburg, T.A.Van De Sluis, BartEtebari, KayvanMiddeldorp, Christel M.Van Den Bergh, HubertEtienne, LucieMidgley, AdamVan Den Meiracker, Anton H.Etnier, Jennifer L.Miele, ClaudiaVan Der Heiden, KimEuler, GerhildMigliaccio, Anna RitaVan Der Kamp, Marc W.Evangelisti, EdouardMiguel, GraçaVan Der Kuyl, AntoinetteEvangelou, NikosMiguel, Leiva-BrondoVan Der Schee, Marc PhilippeEvans, DavidMiki, YasuhiroVan Der Spoel, Aarnoud C.Evans, Joseph L.Mikoshiba, KatsuhikoVan Der Veer, Eric P.Evans, MalkanthiMilic, NatasaVan Dijk, Bart G.M.Eve, David J.Milione, MassimoVan Etten, JamieEvensen, ØysteinMillecamps, MagaliVan Hemelrijck, MiekeEverts, HelenMilledge, John J.Van Leeuwen, J.Every-Palmer, SusannaMiller, Aaron W.Van Leeuwen, ThomasEyers, ClaireMiller, AlysonVan Munster, Jolanda M.Ezendam, JanineMiller, JohnVan Oers, NicolaiEzura, YoichiMilling, SimonVan Putten, Michel J.A.M.Fabiani, RobertoMilner, Caroline M.Van Raaij, Mark J.Fabregat, IsabelMilner, JoelVan Rijn, Richard M.Facchini, GaetanoMimaki, YoshihiroVan Werkhoven, Cornelis H.Fadl, AminMimura, TetsuroVan Westering, Tirsa L.E.Faehling, M.Minagar, AlirezaVandenwijngaert, SaraFagerstedt, KurtMinakata, DaisukeVandersteen, AnthonyFahraeus, RobinMinami, IchiroVanderWeele, DavidFaísca, PatríciaMinarchick, ValerieVandooren, JenniferFalk, HeinzMinbiole, Kevin P.C.Vanella, LucaFalk, Roni T.Mindt, Thomas L.Vapaatalo, HeikkiFalone, StefanoMine, YuichiVaporidi, KaterinaFan, FengjuanMinelli, AlbaVaradarajan, SridharFan, XiuchengMingorance, JesúsVarallyay, EvaFañanas, Francisco J.Minor, PhilipVarela-López, AlfonsoFang, BaiMiquel, Barceló-OliverVargiu, Attilio V.Fang, JianwenMiranda, CarlosVashee, SanjayFang, YeMiron, Richard J.Vashist, Yogesh K.Fanucci, Gail E.Mirzayans, RazmikVasquez, ElisardoFarber, Steven ArthurMisevic, Gradimir N.Vassilopoulos, GeorgeFarinos, H. MerleMishra, NigamVatta, MatteoFaroni, AlessandroMishra, Yogendra KumarVaughn, ByronFarrow, KathrynMishur, Robert J.Vaz, Deisi AltmajerFaul, ChristianMisra, Biswapriya B.Vecchione, CarmineFavas, PauloMisu, TatsuroVecoli, CeciliaFedder, JensMitani, AkioVedel, Anne G.Fedele, FrancescoMitchell, Scott G.Velasco Ortega, EugenioFedorova, Olga V.Mitra, AbhisekVeldhuizen, EdwinFei, LikunMitra, RajaVeleeparambil, ManojFeige, JeromeMitsunaga, ShuichiVelkov, TonyFeinstein, PaulMittnacht, SibylleVelliyagounder, KabilanFelder-Schmittbuhl, M.-P.Miura, NobuhikoVelotti, FrancescaFelger, J.C.Miyagawa, S.Venditti, AlessandroFeliers, DenisMiyake, SachikoVenegas-Calerón, MónicaFeng, YibinMiyake, ShiroVenkatesh, JelliFeng, YuMiyamoto, KojiVenkatesha, ShivaprasadFeng, Z. VivianMiyamoto, TakeshiVenkitachalam, SrividyaFeo, FrancescoMiyamoto, ToshinobuVenter, RietieFeraco, AlessandraMiyamoto, YoheiVenturi, FrancescaFeresin, Rafaela G.Miyata, JunVenuti, AldoFerlizza, EneaMiyata, YasuyoshiVerdoucq, LionelFernandes, EduardaMiyazaki, DaigoVergara, DanieleFernández Robredo, PatriciaMiyazaki, TeruoVergauwen, LuciaFernández, Francisco J.Miyazaki, TetsuroVerhasselt, BrunoFernández, LucíaMiyoshi, DaisukeVerhoeven, Adrie J.Fernandez, MichaelMiyoshi, EijiVerrecchia, FranckFernandez-Botran, RafaelMiyoshi, HiroakiVerri, TizianoFernández-García, M. IsabelMiyoshi, NorioVerron, EliseFernandez-Garcia, MartaMizoguchi, ToshihideVerstuyf, AnnemiekeFernandez-Lafuente, RobertoMizuo, KeisukeVetvicka, VaclavFernandez-Lima, FranciscoMo, HuanbiaoVicente-Carrillo, AlejandroFernandez-Palomo, CristianMoal, IainViejo-Borbolla, AbelFernandez-Pozo, NoeMobed-Miremadi, MaryamViel, Tânia AraújoFernández-Ruiz, JavierMocellin, SimoneViennois, EmilieFernie, AlisdairModirrousta, MandanaVierra, CraigFerrand, JonathanMoeller, Hanne B.Viggiano, DavideFerrandino, IdaMoffet, DavidVignard, JulienFerrante, AntonioMogi, MasakiVik-Mo, Einar OslandFerraresso, MarianoMoham, AzaVilanova, ManuelFerrero, DarioMohamed, RiyazVillanneau, RichardFerreti, ClaudioMohammed, SulmaVinciguerra, ManlioFerretti, ClaudioMohanam, SanjeevaVink, RobertFerretti, ElisabettaMohanram, H.Vinnedge, Lisa PrivetteFerrieres, VincentMohanta, TapanViruel, Maria AngelesFerrio, J.P.Mohanty, Smruti R.Visioli, FrancescoFerroni, PatriziaMöhlendick, BirteVitetta, LuisFields, Gregg B.Mohn, DirkVitorino, RuiFilella, XavierMolasiotis, AthanasiosVittori, SauroFilep, János G.Moldzio, RudolfVittorioso, PaolaFiligheddu, NicolettaMöller, HeikoVizan, PedroFilosto, MassimilianoMolnar, FerdinandVladimirov, Vladimir I.Fine, James BurkeMolyneux, KarenVlase, LaurianFinelli, CarmineMonaco, Edward A., IIIVlashi, ErinaFiner, YoavMoncalián, GabrielVodnar, Dan CristianFinkelstein, Jacob N.Monchau, FrancineVoelkers, MirkoFiocco, DanielaMonedero, VicenteVogel, UllaFiona, LynchMoniche, F.Vogelaar, Christina FranciscaFioravanti, AntonellaMonji, AkiraVolk, DavidFiorenza, Maria TeresaMonk, JenniferVolk, MartinFischer, KeelanMonsalve, MariaVolz, David C.Fischer, SilviaMontagné, NicolasVon Brunn, AlbrechtFisher, ScottMonteil-Rivera, FannyVon Eggeling, FerdinandFiume, GiuseppeMontenegro, LuciaVon Knethen, AndreasFlamant, QuentinMontenegro, Maria FVonk, Lucienne A.Flanagan, Sarah P.Montes Resano, MartaVoss, MatthiasFlematti, Gavin R.Montoya, GuillermoVougas, KostasFlex, AndreaMoon, Andrea F.Vuckovic, DajanaFlorczyk, Stephen J.Moon, Il SooVuppalanchi, RajFlorea, Ana-MariaMoore, Robert J.Vyas, MaulikFlores, Francisco B.Moos, PhilipWaalen, JillFlorio, TullioMorales, Juan C.Wabiko, HiroetsuFlower, AndrewMorales, JulioWada, JunFlynn, AidanMorales-Cruz, AbrahamWada, Nikolas I.Flynn, Charles R.Morales-Santana, SoniaWada, RyuichiFoan, Chin ChiewMorán, PalomaWade, ScottFoderà, VitoMoran, YehuWadehra, MadhuriFogarty, GeraldMorandi, LucaWadhwa, SunilFogdell-Hahn, AnnaMorbidelli, LuciaWagenmakers, A.J.M.Fogolari, FedericoMorbidelli, MassimoWaggoner, Stephen N.Folco, Hernan DiegoMoreau, RegisWagner, AnikaFong, Wye-KhayMoreira, António S.Wagner, Brent T.Fontalba-Navas, AndresMoreira, Maria TeresaWagner, Bridget K.Fontana, Robert JMorel, Jean-BenoitWagner, DavidFontecha, JavierMorello, RoyWähälä, KristiinaForbes-Hernandez, T.Y.Morgan, IainWahlestedt, ClaesForini, FrancescaMorgat, ClémentWahli, WalterForlemu, NevilleMorgutti, SilviaWakabayashi, IchiroFormisano, PietroMori, KiyoshiWakamatsu, KazumasaFormosinho, Sebastião J.Mori, MasaakiWakisaka, NaohiroForn-Cuni, GabrielMori, MatteoWaldau, BenForner, StefâniaMori, SeiichiroWaldmeier, FelixForo, PalmiraMori, YoshiharuWalker, William B., IIIForrest, LucyMoriguchi, TakayaWalker, Douglas G.Fort, PatriceMorikawa, ToshioWalker, HeatherForton, F.M.N.Morita, KyojiWalker, JessicaFortunato, OrazioMorley, Steven DouglasWalker, M.M.Fossdal, Carl GunnarMoro, LoredanaWallace, HeatherFoster, DavidMorreau, HansWalsby, Charles J.Foster, Kenneth R.Morrill, Jason A.Walser, Tonya C.Fournier, ClaudiaMorris, Brian J.Walsh, DavidFournier, Pierre-EdouardMorrow, JenniferWalters, DianneFox, SusanMorshead, Cindi M.Walther, DirkFozo, ElizabethMortimer, PeterWan, DehuiFracanzani, Anna LudovicaMoschetta, MicheleWan, XuehuaFraccarollo, DanielaMoschovi, MariaWandosell, FranciscoFradley, Michael G.Moseke, ClausWang, AijunFrago, EnricMosher, M.J.Wang, Bo-ChengFragouli, DespinaMothe, BeatrizWang, Ching-ChiungFrancesca, MangialascheMotorine, IouriWang, Chin-KunFranceschi, PietroMoura, AndreWang, Chrong-ReenFrancino, PilarMouradian, M. MaralWang, ChuanfengFrancis, HeatherMouradov, AidynWang, DanFranco, Aime TMousa, Shaaban A.Wang, DavidFranco, DiegoMoustris, KostasWang, Feng-ShengFranco, RodrigoMu, DezhiWang, HuanyouFrank, StephanMuccilli, VeraWang, Huei-JuFranken, PhilippMühl, HeikoWang, HuiFranzke, Claus-WernerMuinelo, LauraWang, Jeh-JengFraser, Justin F.Muir, KeithWang, JianFreedman, Bruce D.Muise-Helmericks, Robin C.Wang, Jian-WenFreeman, Jennifer L.Mujagic, ZlatanWang, JianyingFreije, José M.P.Mukaida, NaofumiWang, JoshuaFreise, ChristianMukherjea, DebashreeWang, JuanFreking, BradMukherjee, SomnathWang, JunFrench, LarsMüller, CarolineWang, JunboFrenea-Robin, MarieMüller, Christian P.Wang, JunjieFresco, PaulaMüller, DafneWang, KaiFreundt, Eric C.Muller, Patricia A.J.Wang, Kevin YuejuFriedlander, Terence W.Mullin, James M.Wang, LeFroeyen, MatheusMunang’andu, Hetron M.Wang, Ling ZhiFröhlich, EleonoreMungamuri, Sathish-KumarWang, MaxwellFrost, SofiaMunoz-Amatriain, MariaWang, PengFry, Jessica L.Murakami, YasufumiWang, QiFu, JidongMurakami, YoshikiWang, QimingFu, Shih-FengMuralidharan-Chari, V.Wang, QunFuchs, BrunoMurashita, KojiWang, Raymond Y.Fugazzola, LauraMurata, YoshiyukiWang, Robert Y.-L.Fujii, HirofumiMurillo, Ana GabrielaWang, RuiFujii, TakaoMurono, ShigeyukiWang, RuoxiangFujii, TakeshiMurphy, AndrewWang, Shao-ChunFujimori, KoMurphy, Michael E.P.Wang, ShengFujita, MasayukiMurphy, PaulaWang, ShiboFujita, YoshioMurray Stewart, TracyWang, Tian-LiFujitsuka, NaokiMurray, S.A.Wang, Ting-FangFujiwara, RyoichiMurray, Thomas ScotWang, Tzu-WeiFujiwara, Shin-ichiMurugaiyan, JayaseelanWang, Wei-LungFukuda, TakeshiMusch, MarkWang, XiaohongFukuhara, RyojiMusio, CarloWang, XinFukunaga, KenjiMustafa, GhazalaWang, YingFukunaga, KohjiMustafa, SanamWang, YumengFukunishi, YoshifumiMusumeci, GiuseppeWang, Yun-XingFukushima, AtsushiMuthana, MunittaWang, ZhanweiFukushima, TsuyoshiMutoh, MichihiroWang, ZhijieFulkerson, Patricia C.Mutsaers, Steven E.Wang, ZhixiangFuller, BryanMuylaert, Dimitri E.P.Wang, ZhizhiFullston, TodMuylkens, BenoîtWang, ZongweiFulton, Amy M.Myers, Damian E.Wangchuk, PhurpaFumihiko, KatakuraMyers, Daniel D.Wanke, DierkFunaba, MasayukiMyers, Linda K.Warby, Simon C.Fürnsinn, ClemensMysliwiec, TamiWard, Peter A.Furue, MasutakaNachappa, PunyaWary, Kishore K.Furuichi, KengoNadiminty, NagalakshmiWatanabe, MasakatsuFuselli, FabioNadler, Jerry L.Watanabe, MasatoshiFuso, AndreaNaegele, R.P.Watanabe, MasayukiFuster, DanielNaftalin, RichardWatanabe, MineoGaborit, B.Nagai, KouheiWatanabe, YuichiroGabriella, LindgrenNagai, TakayukiWatchko, JonGaerstel, MatthiasNagai, TakeshiWaterhouse, MiguelGafencu, AncaNagai, YoshinoriWaters, MichaelGailani, GaffarNagaraju, Ganji P.Waters, Michael J.Gailhouste, LucNagatoishi, SatoruWaters, PaddyGajofatto, AlbertoNagel, RaimundWatkins, Adam J.Gajula, RajendraNagelhus, Erlend ArnulfWatkins, G. LeonardGal, A.Nagpal, SeemaWatt, SuzanneGalanakis, Charis M.Nagwekar, JanhaviWatzka, MatthiasGaldiero, StefaniaNagy, LaszloWaugh, Mark G.Gales, AmandineNah, Seung-YeolWaxman, David J.Galileo, Deni S.Nahashon, SamuelWaxman, Stephen G.Gall, HenningNajimi, MustaphaWeber, Daniel GilbertGallardo, Rodrigo A.Naka, HiroakiWeber, Franz E.Galli, Dominique M.Nakagawa, ShunsakuWee, Joseph T.S.Gallicchio, EmilioNakagawa, YoshimiWegner, Marthe-SusannaGallie, Daniel R.Nakahama, Ken-ichiWei, AndrewGallo, LindaNakahara, KazuhikoWei, ChangyongGalus, SabinaNakajima, HirokoWei, GanGalván, IsmaelNakaki, ToshioWei, JianjunGambarotta, GiovannaNakamoto, MasatoshiWei, SainanGambino, G.Nakamoto, ShingoWei, Shau-MingGams, WalterNakamura, KazufumiWei, ShengGan, R.Y.Nakamura, MasatoWei, YufengGanea, D.Nakamura, NorikoWeiergräber, MarcoGanguli-Indra, GitaliNakamura, ShingoWeigand, AnnikaGanini, DouglasNakamura, TakahiroWeigand, Julia E.Gantenbein, BenjaminNakamura, TsukasaWeiser, Douglas C.Gao, P.Nakamura, YojiWeisleder, NoahGao, XueNakano, KazuhikoWeljie, AalimGao, ZhiqiangNakano, MiyakoWelsch, RalfGarcia, MichaelNakashima, KazuoWen, C.M.García-Jimenez, IkerNakashima, SouichiWen, XiaominGarcia-Redondo, Ana BelenNakashiro, Koh-ichiWendell, Stacy GelhausGard, PaulNakatani, YoshihikoWeng, Ching-FengGarinis, GeorgeNakatsuka, TakashiWerling, DirkGarner, BiancaNakayama, TakayukiWerner, IngeGarrett, ScottNakayama, YujiWerner, ThomasGarrity, DeborahNakazawa, MasamiWerstuck, Geoff H.Gasparrini, MassimilianoNalam, VamsiWertz, Philip W.Gasser, ChristophNam, Kyoung HeeWestenfelder, ChristofGatti, LauraNanda, HimansuWesthoff, AndrewGaudenzio, NicolasNandi, SaikatWestin, GunnarGaudet, AndrewNandwana, VikasWeström, BjörnGavaia, P.J.Nanjappa, ManjunathaWestwell, AndrewGavira, José A.Nanni, LorisWetzel, ChristianGavish, MosheNaqvi, AfsarWheeler, ThomasGazouli, MariaNarani, AkashWhipple, C.A.Ge, XiaodongNarayana, AshwathaWhisstock, James C.Gee, Christine ENarayanan, AnandWhite, PeterGehrs, Karen M.Narayanasamy, PrabagaranWhite, Thomas A.Gekle, MichaelNardelli, Dean T.Whitehurst, Angelique W.Geldof, LoreNardon, ChiaraWhysall, KasiaGelpi, Josep LluisNarendhirakannan, R.T.Wiborg, OveGémes, KatalinNarod, Steven A.Widgerow, Alan D.Genetos, DamianNarute, PurushottamWie, Myung-BokGenji, ImokawaNascimbeni, FabioWieben, OliverGenovese, GiuseppaNascimento, Alessandro S.Wiese, StefanGensel, John C.Nasopoulou, ConstantinaWigle, J.T.Gentile, PiergiorgioNasrallah, June B.Wijburg, FritsGeorgakilas, Alexandros G.Nass, NorbertWilber, AndrewGeorge, GrahamNassuth, AnnetteWilhelm, StefanGeorge, VargheseNastro, Rosa AnnaWilkins, Laetitia G.E.Georgiev, VasilNatalello, AntoninoWillerth, Stephanie M.Geraci, FabianaNatarajan, Sathish KumarWillför, StefanGerardi, CarmelaNatesan, SenthilWilliams, Andrew R.Gerber, Leonard E.Nathanson, S. DavidWilliams, Bart O.Gerdol, MarcoNavarra, MicheleWilliams, DavidGerhard, MarkusNavarro, Esther PérezWilliams, Jack D.Gericke, MartinNaviglio, SilvioWilliams, JamesGerothanassis, Ioannis P.Naya, FranciscoWilliams, NicoleGershburg, E.Nazar, Ross N.Williams, Thomas L.Geso, MoshiNazaruk, JolantaWilliams, TiffanyGewirtz, DavidNazzal, SamiWilliamson, MikeGhaleh, BijanNde, Divine B.Willison, HughGhandhi, Shanaz AdiNedachi, TakuWilloughby, DarrynGharbia, Saheer E.Negre-Salvayre, AnneWilson, Anthony G.Ghavami, SaeidNelson, Fred R.T.Wilson, Brenda AnneGhia, Jean-EricNemenoff, RaphaelWilson, David B.Ghosh, SujoyNenoi, MitsuruWilson, DouglasGiaginis, ConstantinosNeri, SimonaWilson, HeatherGiampieri, FrancescaNesmelov, Yuri E.Wilson, RichardGianferrara, TeresaNeumann, MartinWinger, QuintonGiannakourou, Maria C.Neureiter, DanielWinkler, David A.Giannattasio, SergioNewman, DavidWirth, MichaelGiannenas, IliasNg, Chau Hsien MatthewWirths, OliverGiannotti, Marina InésNg, Hui WenWise, JohnGiansanti, FrancescoNG, Kevin Tak-PanWitczak, CarolGiblin, Frank J.Ng, Say KongWiteska, MałgorzataGibson, GregoryNguyen, Dan-VinhWitorsch, Raphael JayGibson, JoshuaNguyen, Linh ThuyWitt-Enderby, PaulaGibson, Susan I.Nguyen, PhuongWitting, MichaelGieling, Roben G.Nguyen, Ruby H.N.Wittung-Stafshede, PernillaGieras, AnnaNguyen, Thomas T.Wolfe, AndrewGilbert, NickNi, WeimingWolff, A.S.B.Gilles, ChristineNianiou-Obeidat, IriniWölfler, AlbertGil-Mohapel, Joana M.Nibbs, Robert J.B.Wolosin, J. MarioGilmore, ReidNicholson, John W.Wolschendorf, FrankGilmour, SusanNicholson, Louise F.B.Woltering, ErnstGilson, MichaelNickerson, ScottWong, Boon-SengGimeno, M. ConcepciónNicolaides, TheodoreWong, Chung F.Ginty, FionaNicolás, Francisco E.Wong, Connie H.Y.Giordano, AntonioNicoletti, FerdinandoWong, George K.C.Giorgio, IvanNicoloso, Milena S.Wong, Grace Lai–HungGirgis, Reda E.Nie, XianzhouWong, Ka-ChunGiron, DavidNiederman, Robert A.Wong, Kenneth K.Y.Gismondi, AngeloNierkens, StefanWong, Michale NingGiuliano, MichelaNieto, GemaWong, Nicholas C.Giuseppe, ZampellaNifli, Artemissia-PhoebeWong, Ronald J.Glading, Angela J.Nigatu, Yeshambel T.Wong, Sunny H.Glauser, GaetanNigris, Filomena DeWong, VincentGlavaski-Joksimovic, AleksandraNiidome, TakuroWood, ChristopherGlazer, LilahNiikura, MasahiroWood, Geoffrey A.Glyan’ko, A.K.Nijnik, AnastasiaWooddell, Christine I.Gobbo, OliveroNikamo, PernillaWoodruff, PrescottGoberdhan, Deborah C.I.Nilsson, GunnarWoods, NicholasGoedert, JamesNing, KeWooten, Joshua S.Gohar, EmanNinomiya, KiyofumiWorrall, MargaretGohda, EiichiNishida, NaokiWower, JacekGohla, AntjeNishiguchi, MasamichiWronska, AnettaGöhre, VeraNishimura, KaneyasuWrzaczek, MichaelGoldberg, ErvNishimura, NoriyukiWu, ChuanlongGoldgar, DavidNishimura, TaisukeWu, HaoGolding, Michael C.Nishina, HiroshiWu, HuaizhuGoldoni, MatteoNishitani, ChikakoWu, Jin-YiGoletti, DeliaNishiwaki, HisashiWu, LianfengGollahon, LaurenNishiyama, AkikoWu, Peter K.Goltsov, AlexeyNissen, Peter H.Wu, QianGomes, Ana P.Nistala, RaviWu, Sheng-NanGomes, Maria SaloméNisticò, RobertoWu, Shih-HsiungGómez, Irene CrespoNitulescu, George MihaiWu, Tzong-YuanGomez, NidiaNiwa, RiekoWu, XiaoqingGómez-Florit, ManuelNiyibizi, ChristopherWullaert, AndyGomez-Sanchez, EliseNjoku, Dolores B.Wurtman, Richard J.Gomis, Roger R.Nocentini, GiuseppeWydra, KerstinGonçalves, M. Sameiro T.Nociari, MarceloWymann, MatthiasGonçalves, OlivierNoël, Emily S.Xafenias, NikolaosGonda, Thomas J.Nofer, Jerzy-RochXavier, Nuno ManuelGondhalekar, Ameya D.Noghero, AlessioXia, TianGondi, Christopher S.Noguchi, Constance TomXian, WaGong, FadeNoguchi, KoXiang, DongxiGóngora-Castillo, ElsaNoh, Ji HeonXiang, YuGonzales, Cara B.Nojiri, TakashiXiao, JunhuaGonzalez, JoaquinNomura, M.Xiao, LiGonzalez, PatriceNomura, TakeoXiao, ZhoushengGonzalez, RoserNonami, AtsushiXie, JianpingGonzález-Aguilar, GustavoNonappa, NonappaXie, JinghangGonzález-Bosch, CarmenNonnemacher, Michael R.Xie, MaohuaGonzalez-Conejero, RocioNorman, TrevorXie, QiGonzalez-Reyes, Luis E.Noseda, Diego GabrielXie, ShaojunGonzález-Rodríguez, M.-L.Novack, Gary D.Xie, YangGonzález-Sánchez, M.I.Novák, JanXin, DongyueGoodacre, SaraNovak, RichardXin, MeiGoodman, RichardNowak, MichaelXing, FeiGoossens, NicolasNoyes, Richard D.Xirodimas, DmitrisGopalakrishnan, SingaramNozell, Susan E.Xu, BaoshanGorczynski, PaulNozik-Grayck, EvaXu, HainengGordon, DonnaNtie-Kang, FideleXu, Jian-NongGorgoulis, Vassilis G.Nuevo, MichelXu, KeliGorman, ShelleyNunes, CláudiaXu, PengfeiGorodetsky, RaphaelNunomura, WataruXu, WeiGosetti, FabioNuthikattu, SaivageethiXu, XinGossage, R.A.Nylander, KarinXu, YanyiGoto, KazuoO’Brien, KristenXu, YiruGoto, YukiObata, YasukoXuan, Tran DangGoua, MarieObermair, Gerald J.Xuan, XiangchunGougelet, AngéliqueObermüller, NicholasXue, XiangGoulielmos, George N.O’Brien, NoraYachida, ShinichiGovaere, OlivierOccelli, FlorentYadav, PankajGowans, EricOcchialini, AlessandroYaegashi, HajimeGozes, IllanaOchiai, TsuyoshiYagi, AkiraGraber, JudithOchiya, TakahiroYagi, FumioGradziel, TomO’Connor, JamesYakovlev, Igor A.Graffelman, JanO’Connor, John J.Yamada, ShuheiGraham, JulieO’Connor, Kathleen L.Yamada, SohsukeGraier, WolfgangO’Connor, Timothy P.Yamakawa-Kobayashi, K.Graille, MarcOdriozola-Serrano, IsabelYamaki, KohjiGrammatikos, GeorgiosOertel, MichaelYamamoto, KeiGranato, DanielOertel, R.Yamamura, SoichiroGranot, DavidOgasawara, ToruYamanaka, KojiGranucci, FrancescaOgata, TadanoriYamanishi, KiyofumiGrãos, MárioOgawa, HidehikoYamasu, KyoGrapputo, AlessandroOgbourne, Steven M.Yamato, MasayukiGrasa, LauraOgi, KazuhiroYamauchi, AkiraGrases, FelixOgino, ShujiYamauchi, TakahiroGrassl, Guntram A.Ogris, ManfredYamawaki, HideyukiGratzinger, DitaOh, Daniel S.Yan, BowenGray, ChristopherOh, Dong-ChanYan, LepingGreenberger, Joel S.Oh, Joo YounYan, LiangGreene, Catherine M.Oh, JunYan, LinGreene, Lesley H.Oh, KeimeiYandeau-Nelson, Marna D.Greene, Michael J.Oh, SangtaekYang, Chang-HaoGreenman, JohnOh, YohanYang, Ding-IGreenwood, Michael T.Oh, Yoon SinYang, DongliGregory, Karen J.O’Hara, StevenYang, Hung-WeiGregory, Simon G.Ohe, KenjiYang, Jenq-LinGrether-Beck, SusanneOhm, Jae-BomYang, JianhuaGriffin, WilliamOhmiya, AkemiYang, MihiGriffis, JohnOhnishi, MasatoshiYang, ShengyongGriffith, KevinOhno, ShoYang, Sze-Ming MildredGriffiths, BryanOhri, Sujata SaraswatYang, TaoGriffiths, Leigh G.Ohta, YoshijiYang, Tsung-LinGril, BrunildeOikawa, AkiraYang, Wei-HsiungGrilli, AlfredoOji, YusukeYang, XueGrimes, PearlOkada, TomoyoYang, YananGrimholt, UnniOkada, YukinoriYang, YeGrimm, Elizabeth A.Okayasu, RyuichiYang, YongguangGrimpe, BarbaraOkegawa, TakatsuguYang, Yu-ChiaoGrobe, Justin L.Okello, EdwardYang, YunzeGroenendyk, JodyOklu, RahmiYano, EiziGrogan, GideonOksanen, EskoYano, ShozoGropman, Andrea L.Oku, HiromiYao, JianGrosche, AntjeOkumura, KazuhikoYao, JiayiGross, GideonOkumura, TadayoshiYao, WeiGrossini, ElenaOlabisi, OpeyemiYao, YanhuaGrosso, ClaraOlajide, OlumayokunYao, Zhong-PingGrotta, James C.O’Leary, StephenYarotskyy, ViktorGrubman, AlexandraOleinick, NancyYassin, M.A.Gruell, HenningOlivares-Navarrete, RenéYasuchika, KentaroGrumet, RebeccaOlivecrona, GunillaYasuda, KazukiGrzebelus, E.Oliveira, Gustavo M.S.Yasuo, ShinobuGu, FengOliveira, JoanaYasutomi, YasuhiroGu, HaiweiOliveira, P.A.Yazlovitskaya, Eugenia M.Gu, JianguoOliveira-Ferrer, LeticiaYe, LinGu, WenyiOliver, Brian G.Ye, Sang-HoGualtieri, MaurizioOliver, Francisco JavierYe, ZhouGuan, XinOliver, RichardYeh, Chau-TingGuarino, FrancescoOliver, StefanYeh, Chih-KoGubler, FrankOlivier, MicheauYeh, Jan-YingGude, Veera GnaneswarOlmedillas López, SusanaYeh, Jwu-LaiGuerini, Franca R.Olsen, NancyYeh, Shu-LanGuerlesquin, FrancoiseOlson, Michael E.Yeh, Wei-LanGuerrero, PedoOlson, Mira S.Yeh, Yin-TingGuerrero-Preston, RafaelOltean, SebastianYellen, Benjamin B.Guerrieri, EmilioOltra, ElisaYen, Feng-LinGugliucci, AlejandroOmar, Syed HarisYen, Jeng-HsienGuhathakurta, SubhrangshuOmi, ToshinoriYin, KingsleyGuidi, LuciaOmoruyi, FelixYin, ShiGuignabert, ChristopheOno, AkiraYin, ZhongchaoGuillou, HervéOnyenwoke, Rob U.Yndestad, ArneGuilpain, PhilippeOo, Ye HtunYochum, Gregory S.Gundogdu, OzanOoi, JennyYokoi, KakeruGuo, FenghaiOon, Hazel H.Yokoi, TsuyoshiGuo, LeiOpdenakker, GhislainYokosuka, AkihitoGuo, YanOpitz, JörgYokota, ShinsoGupta, AnkitOppenheimer, Steven B.Yokoyama, TomoyukiGupta, PrachiOrban, LaszloYoneda, ToshiyukiGupta, ShashiOrciani, MoniaYonekura, MasamiGupta, Vijai KumarOrdónez-Mena, José M.Yonekura, ShinichiGupta, Vivek K.Oresnik, Ivan J.Yoo, SoonmoonGupta-Rossi, NeetuOrlandi, AugustoYoo, Tag KeunGürdeniz, GözdeOrlando, GiuseppeYool, Andrea J.Gurevich, Vsevolod V.Orlova, Elena V.Yoon, Do-YoungGururani, MayankOrsi, AndreaYoon, GyesoonGutiérrez Martín, SantiagoOrtega, Angel L.Yoon, Je-HyunGutiérrez-Juárez, RogerOrth, James D.Yorgason, JordanGutschner, TonyOrucevic, AmilaYoshida, HiroshiGuyeux, ChristopheOsakada, KohtaroYoshida, NaokoGuzman-Puyol, SusanaOsaki, MitsuhikoYoshida, TakashiGuzzo, FlaviaOshitari, ToshiyukiYoshihito, YokoyamaHaaparanta-Solin, MerjaOskeritzian, Carole A.Yoshimura, AkihikoHaase, HajoOster, HenrikYoshinaga, KazuyaHäder, Donat P.Otagaki, ShungoYoshino, JunHadjiargyrou, MichaelOthman, Ahmad SofimanYoshitomi, HiroyukiHadjikakou, Sotiris K.Otsuka, FuminoriYoshiyama, HironoriHa-Duong, Nguyet-ThanhOttewell, PennyYou, XiaonaHadwiger, LeeOttmann, ChristianYou, ZongbingHafizi, SassanOtto, Angela M.Youn, BuhyunHagenbuch, BrunoOursler, Merry JoYoung, DavidHaglund, LisbetOvilo, CristinaYousaf, Muhammad NaveedHagstrom, Stephanie A.Owen, CarolynYu, Cheng-ChiaHahnel, SebastianOyoshi, TakanoriYu, Cheng-PingHaider, LukasOzaki, IwataYu, ChengzhongHaider, NorbertOzawa, YokoYu, Chia-LiHains, DavidOzeki, NobuakiYu, Ching-ChingHalberstadt, Adam L.Ozeki, YoshihiroYu, FengqunHalbwirth, HeidiOzen, MustafaYu, JaecheulHall, Carolyn S.Ozturk, CanYu, RichardHall, RebeccaPacak, AndrzejYu, Yan PingHalterman, DennisPace, Betty S.Yu, YinghuaHalushka, MarcPaci, MaurizioYu, ZhanyangHamaguchi, MasahidePacios, LuisYuan, ChunHamamura, K.Padmanabhan, JayaYuan, ShinshengHamanishi, JunzoPadrela, LuisYuan, XueHamilton, James P.Padula, MatthewYuan, ZhaoHamilton, PaulPagano, LucaYuan, ZhihongHamilton, Stuart T.Pai, BalagopalYuasa, S.Hammer, SuntreaPaik, Hyun-DongYue, PatrickYingKitHammond, JohnPajak, PaulinaYue, XiaojingHamwieh, AladdinPalacios, DanielaYuk, Hyun-GyunHan, Dong WookPalacios, José-ManuelYumoto, HiromichiHan, Quan-BinPalazzo, ElisabettaYusuf, NabihaHan, Sang-BaePalenzuela López, J.A.Zabel, Brian A.Han, Yeon S.Palese, PeterZaccai, MicheleHanaoka, MitsumasaPalitti, FabrizioZachut, MayaHanaor, DorianPalmieri, DarioZago, MiriamHanash, Alan M.Palmirotta, RaffaeleZakharian, EleonoraHancock, RobPalomares, Eva RufinoZambrano, ElenaHanda, OsamuPalumbo, CarlaZamyatnin, AndreyHankey, PamelaPan, Hung-ChuanZandi, KeivanHano, ChristophePanabieres, FranckZandi, OmidHanus, JakubPanáková, DanielaZanetti, MichelaHappel, AustinPanara, FrancescoZang, LiqingHappel, PatrickPanaro, Maria AntoniettaZanin, LauraHarashima, NanaePanchapakesan, BalajiZaphiropoulos, PeterHaraszthy, Violet I.Panda, Amaresh C.Zaramella, PatriziaHarauz, GeorgePandolfi, AssuntaZarbin, MarcoHardeland, RudigerPanee, JunZaunders, JohnHarding, IanPaneni, FrancescoZavattaro, ElisaHardingham, Jennifer E.Panfoli, IsabellaZavitsas, AndreasHardman, W. ElainePang, Myung-GeolZawilska, Jolanta B.Harduin-Lepers, AnnePani, BibhusitaZehbe, KerstinHardy, JohnPanicker, RajeshZehl, MartinHare, Joshua M.Panico, AntonioZeller, SebastianHargrove, MarkPanizzutti, BrunaZemel, MichaelHäring, Hans-UlrichPanse, Rozen LeZempleni, JanosHarmsen, Hermie J.M.Panzella, LuciaZenclussen, AnaHaro, DiegoPao, Ching-IZeng, HuaqiangHarpke, DörtePaolocci, NazarenoZerbini, GianpaoloHarris, Andrew R.Papaccio, GianpaoloZeugolis, DimitriosHarris, Jennifer W.Papadakis, RaffaelloZgaga, LinaHarris, L.K.Papadimitriou, VassilikiZhang, BinHarris, PhilipPapadimitriuou, K.Zhang, CaiguoHarris, Randall E.Papadopoulos, PetrosZhang, ChristieHarrison, Lisa M.Papenbrock, JuttaZhang, DeqiangHarroun, ThadPapi, AlessioZhang, DonglinHarscoat-Schiavo, ChristellePapke, Roger L.Zhang, DuoHartmann-Petersen, RasmusPaquete, Catarina M.Zhang, JianpingHarty, RonaldParagas, NealZhang, JieHarvey, RuthParahuleva, Mariana S.Zhang, JunzengHasegawa, KoichiParakhonskiy, B.V.Zhang, LiHashimoto, MasayukiParakhonskiy, BogdanZhang, MingliangHäsler, RobertPardo, MaríaZhang, PengHassan, MohamedParedes, EstefaníaZhang, QiuyangHassouna, RimParinandi, Narasimham L.Zhang, RuiqinHata, AaronPark, Hyun-WooZhang, TaoHatano, TsutomuPark, JiyangZhang, WeiHatfield, Ronald D.Park, Jong-HoonZhang, WenhengHattori, ToshioPark, JungZhang, WenjunHattori, YuichiPark, Ky YoungZhang, XinHatzopoulos, PolydefkisPark, Seong YongZhang, XuanHaukaas, TonjePark, TaehwanZhang, XuchenHaukka, KaisaPark, YongKeunZhang, Yan-BoHausner, GeorgPark, Yong-KooZhang, YanpengHautbergue, GuillaumeParker Lemieux, KiTaniZhang, YixiangHawes, T.C.Parker, CarolineZhang, YixuanHawinkels, LukasParker, DaneZhang, YuanyuanHayakawa, TohruParkes, MariaZhang, ZhengangHayward, S. DianeParks, WilliamZhao, LiangHazane-Puch, FlorenceParletta, NatalieZhao, MeixiaHazell, Alan S.Parola, MaurizioZhao, NingningHazlehurst, JonathanPartanen, J.Zhao, QianHe, XiaoPartonen, TimoZhao, XiaoaiHe, XiaojuanPasini, LuigiZheng, ShilongHe, ZhenPaslakis, GeorgiosZheng, YueHearn, Michael J.Passarella, DanieleZheng, Zhi-MingHebbard, L.Passi, AlbertoZhong, TuhuaHebelstrup, Kim H.Pastor, FernandoZhou, Donghua H.Hébert, TerryPastores, Gregory M.Zhou, JiaHecker, MichaelPastori, Ricardo L.Zhou, QingxiangHedemann, Mette SkouPatel, Daxesh P.Zhou, WenchaoHedfalk, KristinaPatel, KeyurZhou, XiaomingHedhammar, MyPatel, NiketaZhou, ZhengpingHedley, PeterPatel, Yashomati M.Zhu, FangHeegaard, Niels H.H.Pathak, HarshZhu, HailongHefferon, Kathleen LauraPatil, Sunil A.Zhu, Hai-LongHegazi, Refaat A.Pattanaik, SitakantaZhu, JianhuaHegyi, EszterPatterson, AndrewZhu, LiandongHegyi, PéterPaul, ThornalleyZhu, YefeiHehlgans, StephaniePaulmurugan, RamasamyZhu, YuwenHeianza, YorikoPauly, NicolasZhu, ZheHeidari, MohammadPaz-Filho, GilbertoZiman, MelHeideman, WarrenPazzagli, LuigiaZimmer, PhillipHeider, DominikPearen, MichaelZimring, James C.Heise, HenrikePechanova, OlgaZingg, Jean-MarcHelle, FrancoisPecinka, AlesZoja, CarlamariaHeller, E. DanPedersen, AndersZöller, MargotHellwig, MichaelPedersen, LeaZong, Sheng GuoHelm, RichardPedersen, MangorZorzano, AntonioHemley, Sarah JanePedeux, RémyZou, ShengliHemmer-Hansen, JakobPedroza, MesiasZou, XueqingHenke, AndreasPeijnenburg, WillieZucchi, IleanaHeras, JosephPeinado, Juan R.Zuluaga, PaolaHerbst, AndreasPeitzsch, ClaudiaZünkler, Bernd-JoachimHernández-Alcoceba, RubénPelagalli, AlessandraZuo, LiHernández-Castellano, L.E.Pellei, MauraZupko, IstvanHernández-Rodríguez, CésarPelletier, StephaneZusso, MorenaHernández-Rodríguez, JoséPelosi, PaoloZwarthoff, Ellen C.Herr, Deron RaymondPenfornis, PatriceZweckstetter, MarkusHerrero, María JesúsPeng, Chien-FangZytynska, S.E.Hertz, Daniel L.Peng, Robert


